# Flexible Polyurethane Foams from Epoxidized Vegetable Oils and a Bio-Based Diisocyanate

**DOI:** 10.3390/polym13040612

**Published:** 2021-02-18

**Authors:** Angelica Cifarelli, Laura Boggioni, Adriano Vignali, Incoronata Tritto, Fabio Bertini, Simona Losio

**Affiliations:** Institute for Chemical Sciences and Technologies “G. Natta” National Research Council, Via A. Corti 12, 20133 Milan, Italy; angelica.cifarelli@scitec.cnr.it (A.C.); laura.boggioni@scitec.cnr.it (L.B.); adriano.vignali@scitec.cnr.it (A.V.); incoronata.tritto@scitec.cnr.it (I.T.); fabio.bertini@scitec.cnr.it (F.B.)

**Keywords:** bio-polyols, bio-based isocyanate, flexible polyurethane foams, prepolymer synthesis, thermal and mechanical properties

## Abstract

Bio-polyols from epoxidized soybean and linseed oils and caprylic acid or 3-phenyl butyric acid were prepared using an environmentally friendly, solvent-free method evaluating the presence of triethylamine as catalyst. Side reactions, leading to a cross-linking structure with high density, were reduced, introducing the catalyst and properly tuning the reaction conditions. A medium functionality value of around 3 along with a hydroxyl number up to around 90 mg KOH/g, narrow polydispersity index, and relatively low molecular mass up to 2400 g/mol were the experimental targets. From selected bio-polyols and an aliphatic partially bio-based isocyanate, a series of water blown polyurethane (PU) foams was produced, estimating the effect of the chemical nature of substituents in the polyol backbone on the PU properties. The apparent density of the foams was in the range of 79–113 kg/m^3^, with higher values for foams from the aromatic acid. Flexible polyurethane foams with open cell structure from bio-based polyols were obtained, with higher cavity size and lower pore sizes for foams from caprylic acid. The bio-based flexible PU foams showed comparable Young’s moduli (14–18 kPa) and compression deflection values (4.6–5.5 kPa) and exhibited an almost complete recovery of their initial size.

## 1. Introduction

Polyurethanes (PUs) are a major polymer family [1], with a global market estimated at approximately €53 billion (sixth among all polymers). Due to the multiplicity of their structures and versatile properties, PUs can be used in various applications. They are mainly found as foams (rigid or flexible based on closed and open cells, respectively), but also as denser materials (elastomers, thermoplastics, or thermosets). PUs can also be used as blends with other polymers, as coatings, or as composites with fillers [2,3,4,5,6].

Nowadays, in agreement with sustainable development principles, a great number of studies and developments have been realized in the field of bio-based PUs, conventionally synthesized by reaction of polyols with isocyanates [7,8,9]. Vegetable oils have been extensively studied for their use in producing bio-based polyols [10,11]. They are readily available and offer advantages such as easy processing, chemical functionality, and relatively low cost. Except for castor and lesquerella oils, vegetable oils do not bear hydroxyl groups, thus they must be introduced at the unsaturated sites by chemical reactions. Among them, epoxidation reactions followed by ring-opening of the oxirane group have been widely investigated for the preparation of bio-based polyols [12,13,14,15]. These oxirane ring-opening reactions are generally conducted in the presence of ring-opening agents, such as alcohols or inorganic acids [13,14,15,16,17], at high temperatures and for long times because of the sterically hindered location of the oxirane groups inside the fatty acid chain. Moreover, these conditions promote side reactions leading to the formation of cross-linked structures in polyols that impact on the properties of the final products [18,19]. To overcome these issues and to favor the opening of internal epoxy groups in triglycerides, one approach is the use of catalysts, such as bases (e.g., amines), phosphorus compounds (e.g., triphenyl phosphine), metals (e.g., chromium compounds), and cationic catalysts (e.g., Lewis acids) [20]. Among them, amines have been widely investigated as nucleophilic reagents used to open the oxirane ring and show good catalytic effect in vegetable-oil-based epoxy systems [17]. In particular, Pan et al. reported the use of 1,8-diazabicyclo[5.4.0]undec-7-ene (DBU) as a catalyst in polyol preparation from epoxidized sucrose esters of soybean oil ring-opened by several organic acids [17]. Furthermore, Kessler et al. investigated the ring-opening between epoxidized soybean oil and linseed oil and castor-oil-based fatty acids in the presence of DBU and pyridine as catalysts [18].

However, although polyols prepared from vegetable oils offer a variety of new and interesting structures, their use is still limited since polyols from epoxidation/ring-opening reactions mostly contain secondary hydroxyl groups which are much less reactive towards isocyanates than those containing primary hydroxyl groups. Besides, the number of hydroxyl groups is considered a critical point affecting thermal and mechanical properties [21]. Furthermore, vegetable oils with high level of unsaturation lead to polyols with high OH functionality. This will result in an increase in cross-linking densities of the resulting PUs, which could limit or preclude their use in flexible foams that represent two-thirds of all polyurethane applications [22,23,24]. Furthermore, bio-based polyurethanes, in comparison to the petroleum-derived counterparts [25,26], in PU applications have some limitations. This arises from their heterogeneous composition that may vary from geographic area but also from the limited compatibility with conventional polyols and other ingredients in PU formulations.

From the literature, it is evident that investigations have been particularly focused on bio-based polyols. In contrast, there has been comparatively little work on the use of bio-based diisocyanate. However, commercial versions of bio-based diisocyanate are now available. For example, Vencorex Chemicals (Saint-Priest, France) has commercialized an aliphatic diisocyanate named Tolonate™ coming from palm oil. This isocyanate possesses low viscosity, 25% renewable material, and a green carbon content of around 32% [27]. This isocyanate is generally employed as precursor to synthesize epoxy resin for coating applications. Nevertheless, to the best of our knowledge there are no examples related to the preparation of flexible polyurethane foams with Tolonate™.

Thus, we decided to investigate the use of bio-based diisocyanate Tolonate^TM^ in combination with polyols from vegetable oils for the preparation of polyurethane foams. Moreover, an aliphatic diisocyanate could ensure flexibility to foams from vegetable-oil-derived polyols, characterized by relatively low OH equivalent weight [22,23].

In this study, we report the synthesis of an innovative family of flexible bio-based polyurethane foams from bio-polyols and Tolonate™, with good appearance and mechanical properties, estimating the effect of the chemical nature of the substituents in the polyol backbone on the PU properties. Polyols were synthesized from commercial epoxidized soybean and linseed oil without the use of organic solvents by ring-opening reaction of the oxirane with organic acids under mild reaction conditions, thus obtaining polyols with different functionalities. A Lewis base catalyst, e.g., triethylamine (TEA), was evaluated to facilitate the ring-opening of internal epoxy groups of the epoxidized oil and minimize the formation of cross-linked structures to produce less viscous and more processable polyols with narrow polydispersity indices [19,28]. This synthetic pathway not only allows the polyol to gain hydroxyl groups but also allows the customization of polyols with different functionalities. Molecular masses, molecular mass distributions, linkage pathways, and the thermal stability of polyols were evaluated.

A series of water blown polyurethane flexible foams was prepared by greatly replacing conventional synthetic polyols with new bio-polyols from soybean and linseed oils. The influence of the new bio-polyols on the foam morphology (cellular structure, interconnectivity), density, mechanical, and thermal properties were studied as well.

## 2. Materials and Methods

### 2.1. General Considerations

All bio-polyol syntheses were carried out on a double manifold Schlenk vacuum line under nitrogen atmosphere. Epoxidized linseed oil (ELO) with approximately 6.4 epoxy groups per triglyceride and *M*_n_ equal to 1182 g/mol, and epoxidized soybean oil (ESO) with approximately 4.3 epoxy groups per triglyceride and *M*_n_ equal to 1168 g/mol, were supplied by Hallstar (Chicago, IL, USA) and Tecnosintesi Spa (Bergamo, Italy), respectively. Caprylic acid (CA, ≥99%) and 3-phenyl butyric acid (3PBA, 99%) were purchased from Carlo Erba (Milan, Italy). Partially bio-based diisocyanate (Tolonate™ X FLO 100) was kindly provided by Vencorex Chemicals (Saint-Priest, France). Niax Silicone L-537 XF (Momentive, NY, USA) was kindly supplied by Eigenmann and Veronelli SpA (Milan, Italy) and used as silicone surfactant. Polypropylene glycol (PPG, *M*_n_ ~3000 g/mol) was purchased from Merck KGaA (Darmstadt, Germany) and glycerol (≥99%) and triethylamine (TEA, 98%) from Fisher Scientific Company (Hampton, NH, USA). Deuterated solvent, CD_2_Cl_2_, and hexamethyldisilane (HMDS) for NMR measurements were used as received from Merck KGaA (Darmstadt, Germany).

### 2.2. Synthesis of Bio-Polyols in Presence of TEA

Epoxidized oil (100 g) was placed in a three necked flask, equipped with a condenser. TEA (1% of epoxidized oil weight) was added to reaction flask and was stirred at 40 °C for 40 min. Under continuous stirring, organic acid was added to the reactor dropwise (1 drop/s) and the temperature of reaction was gradually raised. Finally, the vessel was put under vacuum for at least 40 min at 110 °C. The epoxy/carboxyl ratio and reaction temperature were set according to data reported in Table 1.

### 2.3. Preparation of PU Foams via Prepolymer Synthesis

PU intermediates were synthesized in a closed vessel at atmospheric pressure under mechanical stirring. Quasi-prepolymers [29] were prepared by first reacting a large excess of a isocyanate (free isocyanate content about 30 wt %) with PPG for 40 min in the presence of dibutyltin dilaurate (0.01 wt %) and silicone surfactant (0.5 wt %), then afterwards the isocyanate-terminated intermediate, that was formed in the previous step, was reacted with bio-polyol adding again a small amount of catalyst (0.01 wt %). The ratio between PPG and bio-based polyol is 1:2.

PU foams (PUFs) were prepared mixing the PU prepolymer, in a pre-heated container, with catalyst, silicone surfactant, glycerol, and diisocyanate by mechanical stirrer at 1400 rpm for 25 s. Distilled water (1.5 wt %) was used as blowing agent. An isocyanate index, that is the excess of isocyanate over the theoretical amount for 1:1 reaction with all active hydrogen-bearing groups expressed in percentage terms, equal to 100 was reached. The expanded PUFs were crushed after foaming and then cured for 2 h in an oven at 60 °C.

### 2.4. Characterization Techniques

The hydroxyl number (OH number, mg KOH/g) of the polyol was determined by the imidazole-catalyzed pyromellitic dianhydride method, according to ASTM D 4274-99. The acid value was determined according to IUPAC 2.201 standard using the indicator method.

^13^C-NMR spectroscopic analysis of the polyols was carried out on a Bruker (Billerica, MA, USA) Avance 400 MHz NMR spectrometer in CD_2_Cl_2_ at 25 °C. HMDS signal was used as internal reference.

Size exclusion chromatography (SEC) analyses were performed on a Waters (Milford, MA, USA) GPCV2000 system, using THF as mobile phase, at 35 °C with a 0.6 mL/min flow. Sample concentration was set at 2 mg/mL and injection volume at 150 µL. The SEC system was calibrated using polystyrene standards.

FTIR spectra were recorded using a PerkinElmer (Waltham, MA, USA) Spectrum Two spectrometer from 4000 to 550 cm^−1^ with a wavenumber resolution of 4 cm^−1^ in total attenuated reflectance (ATR) mode.

Thermogravimetric analysis (TGA) was performed on a PerkinElmer (Waltham, MA, USA) TGA 7 instrument at a scan rate of 20 °C/min under nitrogen atmosphere. TGA and derivate thermogravimetry (DTG) curves were recorded from 50 up to 750 °C.

The viscosity of the polyol was measured using a TA Instruments (New Castle, DE, USA) AR 2000 dynamic stress rheometer at 20 °C with a flow test in the shear rate range between 0.01 and 1000 s^−1^. The rheometer was equipped with a plate and steel cone (diameter of 20 mm, angle of 0.5 ° and truncation gap of 16 µm).

The cream time and free rise time of PUFs were evaluated according to the standard “cup-test” in ASTM D7487-13E1 using a digital timer. Each test was conducted repeatedly at least five times to minimize experimental error.

A stereo microscope model Solomark-Science (Yuyao, China) Lab 3D Scope equipped with a digital camera was used to characterize the morphology of foams. The apparent density of foam samples was measured according to EN ISO 845:2006.

Mechanical properties of the foams were analyzed through compression force deflection tests performed by a Zwick-Roell (Ulm, Germany) Z010 mechanical testing machine with a load cell of 2.5 kN. Cylindrical-shape specimens (height of 25 mm and diameter of 50 mm) were firstly pre-compressed twice to 75% of their initial height at 250 mm/min.

After a rest of 6 min, the height of each specimen was determined applying a load of 140 Pa. Finally, the specimens were compressed to 50% of their height at 50 mm/min and the final load after 60 s was recorded. Reported data were averaged on three tests per sample.

## 3. Results and Discussion

### 3.1. Synthesis of Bio-Polyols

Renewable bio-polyols with several functionalization degrees were prepared from two different no-food vegetable epoxidized oils coming from soybean (ESO) and linseed (ELO) oil, with approximately 4.3 and 6.4 epoxy per triglyceride, respectively.

**Scheme 1:**
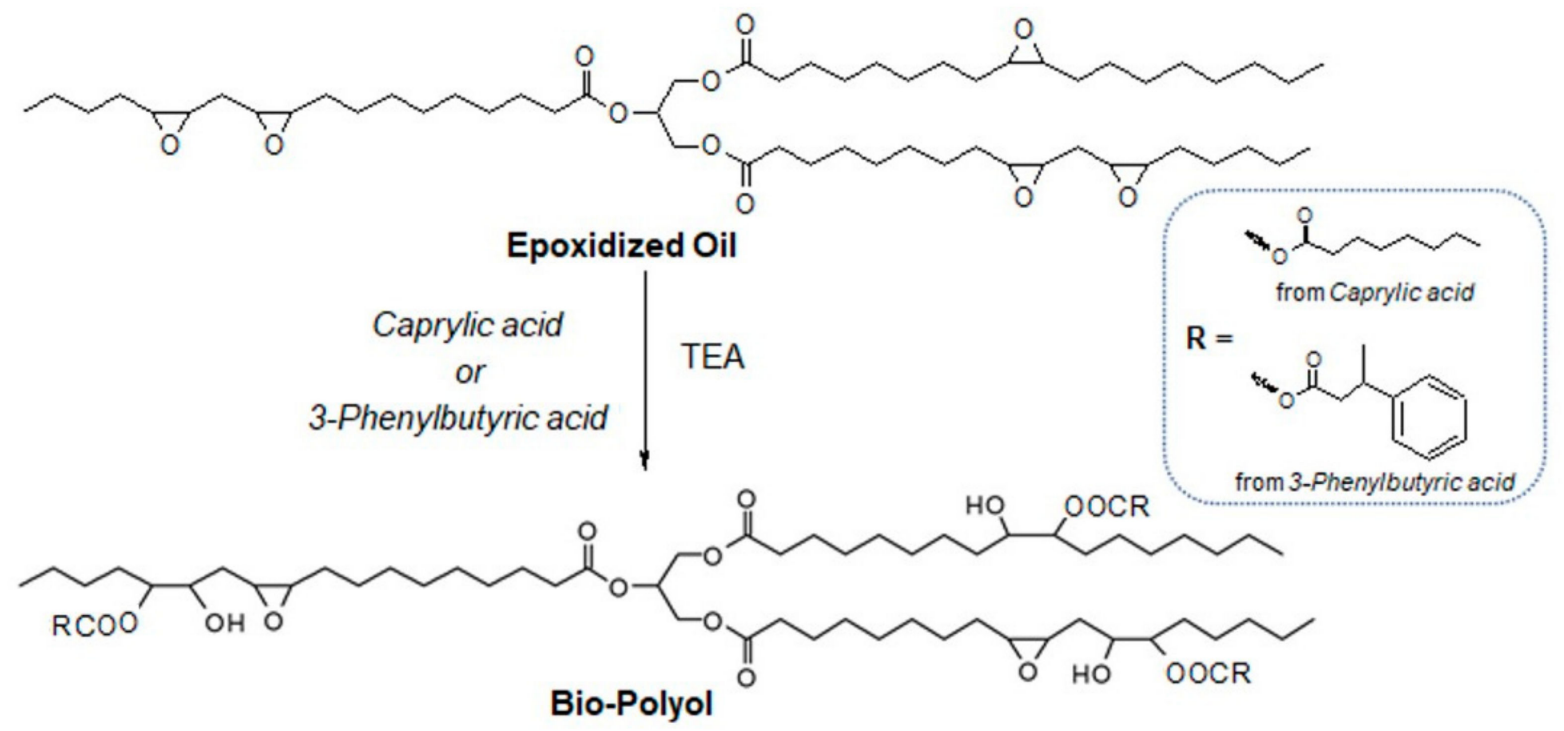
Schematic representation of the synthesis of bio-polyols in the presence of catalyst.

Bio-based polyols were developed using an environmentally friendly, solvent-free method in which no purification or separation steps are required (Scheme 1). Two structurally different organic acids, caprylic and 3-phenylbutyric acid, respectively, were chosen to analyze the effect of an aliphatic short linear chain or the presence of an aromatic motive on PU formulation and properties.

It is worth noting that the hydroxyl functionality of bio-polyols is a well-known key issue for the synthesis of PUFs. With a complete conversion of epoxide group per triglyceride, a maximum total number of OH groups was estimated around 4–6. However, with these high functionalities, rigid foams are expected [29,30,31]. A fine tuning of the reaction conditions in polyols synthesis could provide polyols with relatively high hydroxyl equivalent weight, attractive for the synthesis of flexible PUFs. For our purpose, a medium functionality value of around 3 along with a hydroxyl number up to around 90 mg KOH/g were the targets.

The effects of the epoxy/carboxyl ratio, reaction time, and reaction temperature on the final polyols’ structure and functionalities were investigated (Table 1). The reactions were conducted both in the presence and in the absence of TEA, a Lewis base catalyst. ESO and ELO present several internal epoxy groups, thus the ring-opening reactions are more difficult than in structures with terminal epoxy groups, due to their steric hindrance.

TEA was chosen to facilitate the ring-opening of the internal epoxy groups of the epoxidized oil, while preserving other functional groups of the epoxidized oil, such as triglyceride ester skeletons. Moreover, this catalyst is a well-known catalyst to produce PUFs, thus it is not necessary to remove it after the first step, making this approach both time- and cost-effective.

TEA was also evaluated to minimize the formation of cross-linked structures to produce less viscous and more processable polyols with narrower polydispersity indices that are more suitable for flexible polyurethane foam synthesis. A similar approach was reported in the literature [18] by using DBU as catalyst for the ring-opening reaction and subsequent preparation of polyurethanes.

A careful screening was conducted with ESO and caprylic acid without catalyst (runs 1–3) at 170 °C for 16 h at different epoxy/carboxyl ratios.

**Figure 1:**
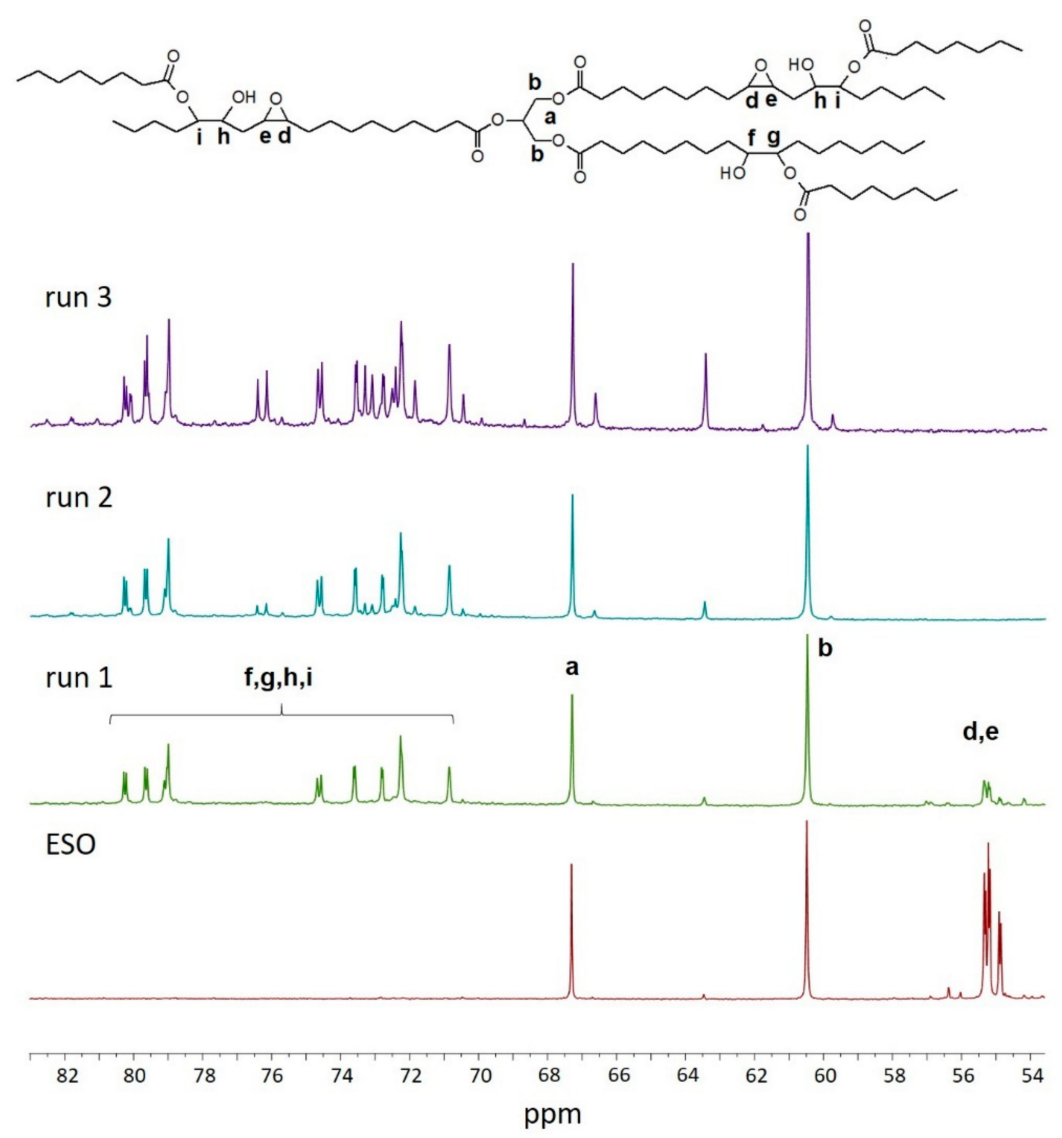
^13^C-NMR spectrum of bio-polyol from ESO (epoxidized soybean oil) and caprylic acid obtained at different epoxy/carboxyl molar ratios. Signals are normalized in respect to signals at 67.3 and 60.5 ppm (methylene and methine carbon atoms of glycerol).

The ^13^C-NMR spectra are shown in Figure 1. This figure shows the region ranging from 84 to 53 ppm; this spectral window was chosen because it is diagnostic of the presence of the resonances of *d* and *e* carbon atoms assigned to epoxy group, in the figure at 56.5–55.0 ppm, and of *f, g*, *h* and *i* carbon atoms in α position to the secondary hydroxyl group and the ester group (81.5–71.0 ppm). Moreover, in this region, resonances at 67.4 and 60.5 ppm are easily assigned to methine and methylene carbon atoms of glycerol, labelled as *a* and *b* carbon atoms, respectively. Only the polyol obtained at epoxy/carboxyl ratio equal to 1:0.5 shows resonances assigned to residual epoxy groups in the region from 55.0 to 56.5 ppm, thus a full conversion of epoxide groups into ester and hydroxyl groups was not achieved at low acid concentration. At increasing acid concentrations, resonances assigned to methylene carbon atoms linked to the hydroxyl group and ester group become preponderant. At high acid concentrations (runs 2 and 3), resonances at 177–178 ppm (not shown) can be assigned to the presence of unreacted caprylic acid in the end product. The ^13^C-NMR analysis showed that at a decreasing epoxy/carboxyl ratio, a more extensive grafting of caprylic acid on oil backbone is achieved.

**Figure 2:**
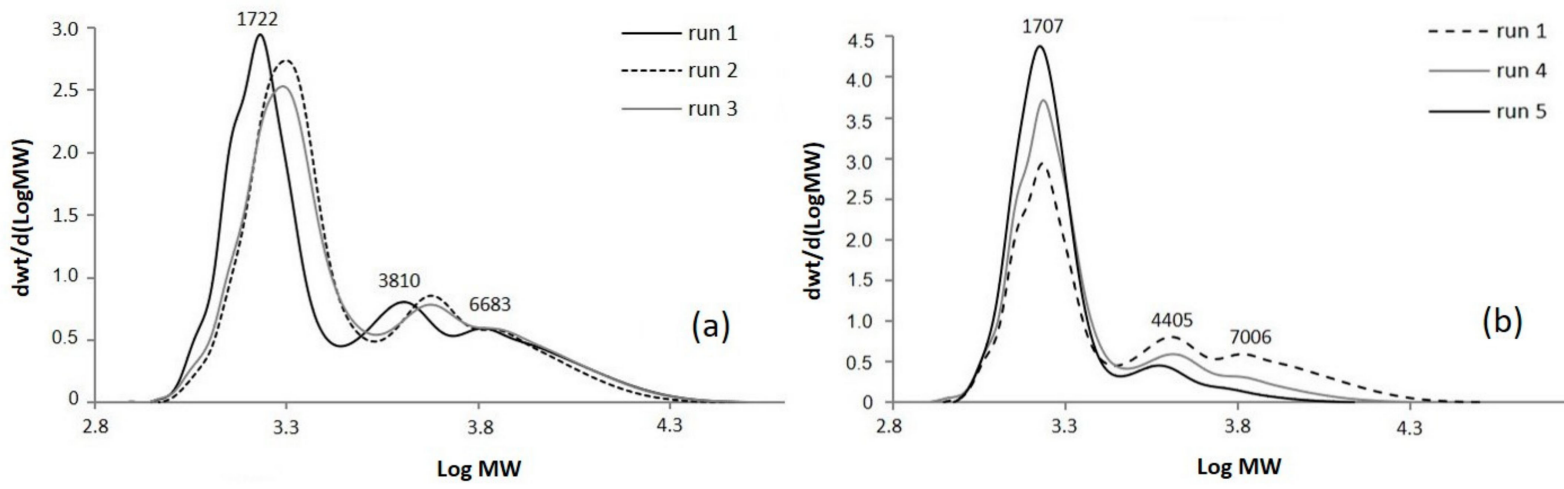
SEC (size exclusion chromatography) chromatograms of bio-polyols from ESO and caprylic acid: (**a**) with different epoxy/carboxyl ratio and (**b**) effect of the presence of TEA (triethylamine) on molecular weight.

In general, comparable molecular weights were achieved (Table 1). The SEC curves as function of molecular weight (MW) in Figure 2 show, for all the samples, a main fraction, whose molecular weight depends on the functionalization degree of epoxidized oil, and minor fractions at higher molecular weight which can be ascribed to by-products with a variable number of grafted side chains (Figure 2a). This oligomeric fraction, present in all the polyols at about 3800–5400 and 6500–7000 g/mol, corresponds to trimers and tetramers respectively, with an ester functionalization degree between 2–3 per oil molecule.

A low epoxy/carboxyl ratio does not avoid the cross-linking side reactions that lead to increased OH functionality and concurrently lead to residual unreacted acid in polyols reaching up to 13% (ratio 1:0.75) and 23% (ratio 1:1) as it is estimated from acid numbers reported in Table 1 (runs 2 and 3). Moreover, the excess of unreacted acid should be reduced for obtaining consistent polyurethane foams.

At a 1:0.5 epoxy/carboxyl ratio, some reactions have been conducted with TEA as catalyst (runs 4–7) and the corresponding SEC chromatograms are shown in Figure 2b and Figure S1 Supplementary Materials. The SEC curve of run 4 highlights a narrower molecular weight distribution with a decrease of the oligomerization peaks (*M*_w_ at 4405 and 7006 g/mol, respectively) when compared to reference run 1, carried out in the same experimental conditions (Figure 2b). This can be explained by the fact that TEA hinders ring-opening by hydroxyl groups, limiting oligomerization. When the reaction was conducted in presence of TEA at reduced temperature (130 °C, run 5), 87 wt % of no-oligomerized oil was achieved, with only the 10 wt % of the trimer. These results point out how the presence of TEA strongly influences the ring-opening and cross-linking reactions, disfavoring the formation of the oligomers. Moreover, when TEA is used, the reactions can be carried out at low temperatures obtaining an OH number around 87 mg KOH/g, comparable with that obtained at 170 °C (run 5 and run 4).

Reducing the reaction time from 16 to 6h and running at 110 °C (run 6), a less extensive ring-opening reaction was achieved than run 5, as shown by shoulder due to unreacted vegetable oil (*M*_w_ corresponding about to 1200 g/mol) (see Figure S1 Supplementary Materials). Moreover, the low reaction temperature did not avoid the formation of a significant oligomeric fraction which consists of about four oil molecules (19% of total chromatogram area). The high hydroxyl number, recorded for run 6, could be explained by side reactions that lead to increase -OH functionality.

Finally, ESO and caprylic acid were reacted at 150 °C for 6 h (run 7) obtaining an adequate end product. Nevertheless, evaluating the residual acid value, the OH number, the molecular weight, and the polydispersity index, the best bio-polyol from ESO and caprylic acid resulted from run 5, carried out at 130 °C for 6 h, at 1:0.5 epoxy/carboxyl molar ratio and in the presence of TEA.

**Figure 3:**
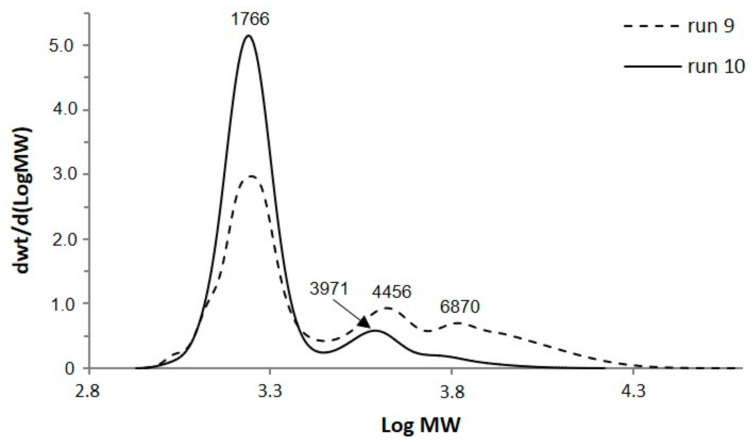
SEC chromatograms of bio-polyols from ESO and 3-phenyl butyric acid.

3-Phenyl butyric acid was used to study the effect of aromatic moieties of bio-polyols on the PUF synthesis, properties, and structure. The reactions with ESO were first conducted at 150 °C (run 9) then afterwards adopting milder temperature (110 °C) in the presence of TEA (1 wt %). As can be observed in Figure 3, the presence of TEA along with a mild temperature leads to a better control of PDI (1.1 vs 1.6 when TEA is absent) and to lower amount of oligomers. The first oligomeric faction whose molecular weight corresponds to 3970 g/mol was ascribed to low substituted trimer (or to high substituted dimer) of soybean oil; it was about 12% of total chromatogram area for run 10. The second oligomeric faction for run 10, whose molecular weight corresponds to 6150 g/mol, was only 2% of total chromatogram area, was ascribed to the trimer of soybean oil with 2.5 acid molecules grafted per soybean oil unit.

As already observed for bio-polyols from ESO and CA, the reactions conducted at low temperature in the presence of TEA lead to OH values in the desired range.

Finally, two reactions were carried out using ELO (run 8 and 11 in Table 1). Some preliminary trials performed at 150 °C overnight with different epoxy/carboxyl ratios (from 0.5:1 to 2:1) showed that this epoxidized oil tends to favor side reactions in comparison with ESO leading to more extensive oligomerization and consequently, higher molecular weights and polydispersity indices. Thus, this higher reactivity of ELO towards the self-polymerization was partially balanced with a short reaction time (4 h) and low temperatures, 150 °C for CA and 130 °C for 3PBA, giving rise to 20% of oligomer content in the bio-polyols from ELO. Furthermore, in this case, the 1:0.5 epoxy/carboxyl ratio represents the most promising conditions to balance the functionalization degree and amount of unreacted acid in the end product.

**Figure 4:**
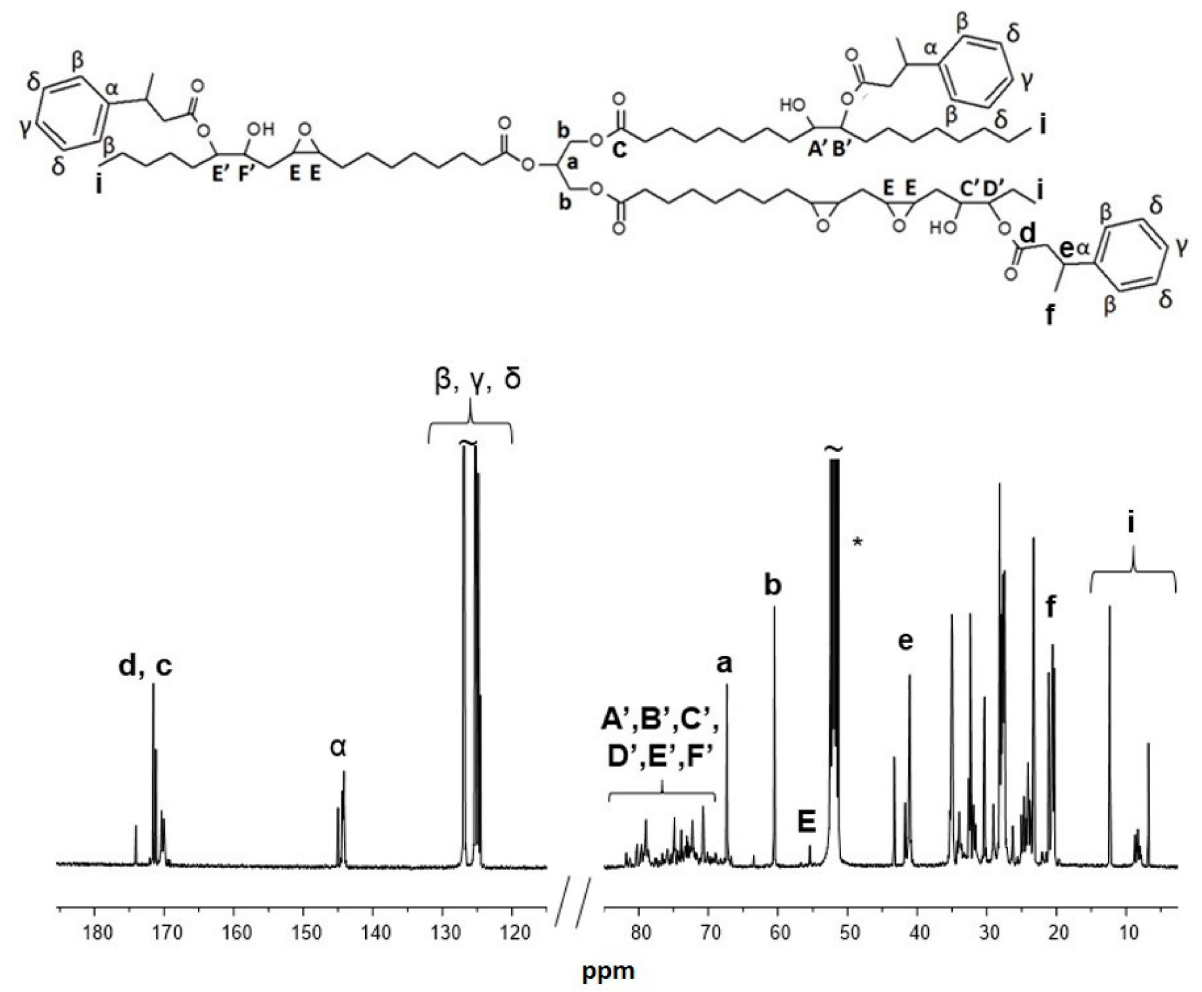
^13^C-NMR spectrum of bio-polyol from ELO (epoxidized linseed oil) and 3-phenyl butyric acid (run 11 in Table 1). * CD_2_Cl_2_ was used as solvent.

^13^C-NMR analysis clearly shows the grafting of caprylic and 3-phenyl butyric acid on the ELO and ESO backbone due to the presence of the characteristic resonance belonging to carbon atoms in α position to the secondary hydroxyl group and the ester group (see Figures S2–S4 Supplementary Materials). Herein, the ^13^C-NMR spectrum of bio-polyol from ELO and 3-phenyl butyric acid is shown in Figure 4. The assignment was conducted according to the literature [32] and by comparing the spectrum with those of the epoxidized oil and of the corresponding organic acid.

Typical resonances of the glycerol structure can be easily assigned: the methine carbon atom labelled as *a* is observed at 69.1 ppm, the methylene carbon atoms in α position with respect to the methine (carbon *b*) appear at 61.9 ppm, while carbon *c* of the carboxylic acid group falls in the region from 171.1 and 171.6 ppm. The methylene groups showed chemical shifts in the range between δ 21.0–36.00 ppm, due to adjacent groups’ effect, and their position with respect to each functional group. Resonances from 12.20 to 8.0 ppm are observed and assigned to the carbon atoms of the methyl groups of the main chain.

Carbon atoms labelled as A’, C’, F’ and B’, D’, E’ in α position to the secondary hydroxyl group and the ester group, respectively, showed resonances in the region ranging from 81.5 to 71.0 ppm. The residual epoxy groups showed resonances in the region from 55.0 to 56.5 ppm, thus confirming the partial conversion of epoxy group, in line with the results from alcohol titration.

The carbon atoms of the aromatic moieties showed typical resonances in the region from 124.0 to 127.0 ppm (carbon atoms labelled as β, δ and γ) and at 144 ppm for carbon α. The carbon atoms of the carboxyl acid group from the aromatic linkers (d) showed a resonance signal at δ 170 ppm, in the same region of carbon c.

As mentioned above, a medium functionality value of about 3 along with a hydroxyl number up to 93 mg KOH/g were considered suitable to prepare polyurethane prepolymers. Moreover, the 1:0.5 epoxy/CA ratio seemed the most promising compromise for obtaining this functionalization degree with a negligible amount of unreacted acid. Thus, bio-polyols obtained from run 5, 10, 8, and 11 with hydroxyl number ranging from 67 to 93 were chosen to prepare PU foams.

The complete characterization of the chosen bio-polyols is reported in Table 2.

The acid content in all bio-polyols was below 20 mg KOH/g confirming organic acid grafting on vegetable oil backbone. Lower acid values (2–3 mg KOH/g) were observed for bio-polyols from caprylic acid with respect to those observed for bio-polyols from 3-phenyl butyric acid with acid value between 15–20 mg KOH/mg, corresponding to maximum 10 wt % of unreacted acid in end product. It is worth noting that these residual acidities did not interfere with the polyurethane synthesis.

Moreover, it was found that polyol from run 8 had the lowest OH functionality although the low acid number demonstrated almost complete caprylic acid grafting on the vegetable oil backbone. However, a portion of oil was not converted into polyol as shown by the shoulder corresponding to 1200 g/mol in SEC chromatogram of run 8 (see Figure S5 Supplementary Materials).

In general, the viscosity of bio-polyols is correlated with the number of cross-linked structures and unreacted acid [19]. Furthermore, the side chains linked to the backbone of the polyols influence the rheological behavior as well. All the characterized bio-polyols showed a behavior typical of Newtonian fluids exhibiting a constant viscosity in the whole range of strain rates investigated (see Figure S6 Supplementary Materials). The introduction of aromatic moieties from 3-phenyl butyric acid leads to an increase in viscosity (run 10 and 11 in Table 2) since the presence of the aromatic ring increases the rigidity of the side-chain and the intermolecular friction resistance [33]. In particular, the viscosity of these bio-polyols increases by one and two orders of magnitude, respectively when compared with that of polyols from caprylic acid. These enhancements in viscosity could be related to the different molecular weight distributions between run 10 and 11 [34]. In fact, observing the SEC chromatograms of both samples it emerges that the fraction corresponding to 3900–4100 g/mol represents 16% and 12% for run 11 and 10, respectively. The main fraction in run 11 corresponds to higher molecular weight (about 1900 g/mol) than in run 10 (1750 g/mol), (see Figure S5 Supplementary Materials), arising from more extensive functionalization by organic acid, according to OH titration (Table 2). At the same time, the functionalization of oil could involve multiple adjacent sites on the same aliphatic chain in linseed oil, that contains a larger amount of linolenic acid (50–55%) than in soybean oil chain (13%) [35,36]. This could increase the viscosity due to steric hindrance, arising from packed aromatic substituents on the same side chain of oil, limiting the molecules flow for the bio-polyol obtained from run 11.

**Figure 5:**
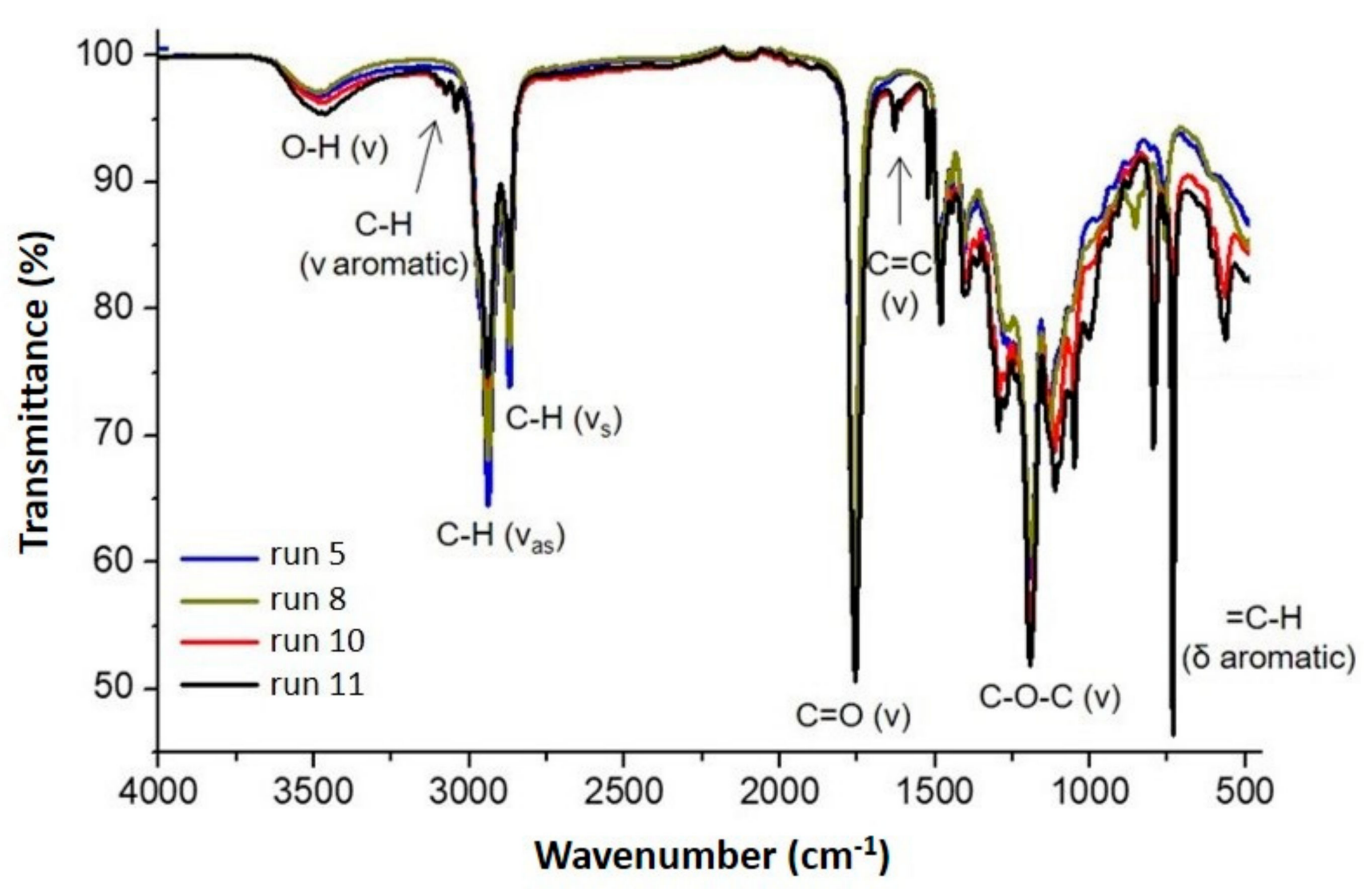
FTIR spectra of the selected bio-polyols from CA (caprylic acid; run 5, 8) and 3PBA (3-phenyl butyric acid; run 10, 11).

The FTIR spectra of the selected bio-polyols for PU synthesis are presented in Figure 5. The broad absorption peak between 3600–3200 cm^−1^ for all spectra is characteristic for stretching vibrations of OH groups, C=O stretching vibration of esters at 1738–1732 cm^−1^, and –CH_2_ symmetric and asymmetric stretching at ~2924 and 2854 cm^−1^, respectively [18]. The oxirane ring vibration peak at 824 cm^−1^ of epoxidized vegetable oils was weakly present according to ^13^C-NMR analysis. Moreover, polyols from 10 and 11 runs showed typical absorption peaks of C–H stretching at 3009–3088 cm^−1^, C=C stretching at 1585–1603 cm^−1^ and =C–H bending at 699 and 762 cm^−1^ of monosubstituted aromatic groups from 3-phenyl butyric acid.

**Figure 6:**
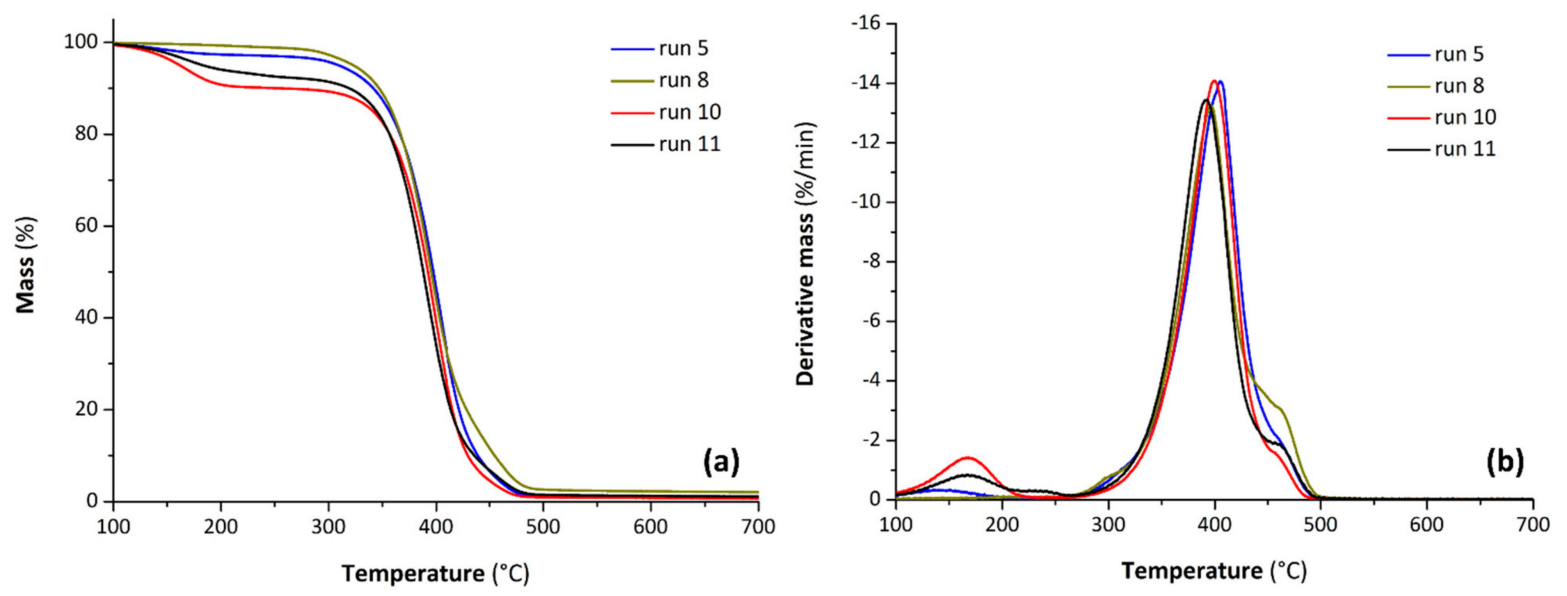
TGA (thermogravimetric analysis) (**a**) and DTG (derivate thermogravimetry) (**b**) curves for selected bio-polyols from CA (run 5, 8) and 3PBA (run 10, 11).

Figure 6a,b show the TGA and DTG thermograms of the selected bio-polyols, respectively. The samples prepared from CA exhibit a good thermal stability and a temperature of degradation at 5% mass loss over 300 °C. Regarding the samples prepared from 3PBA, a first degradation with a loss mass between 8% and 10% attributed to the presence of unreacted acid at lower temperature is well highlighted by a DTG peak centered at about 170 °C. The main degradation process of the bio-polyols takes place between 300 and 500 °C with a corresponding DTG peak centered at ca. 400 °C for each sample. Moreover, all the analyzed polyols present a residual mass at 700 °C in nitrogen approximately of 1%.

### 3.2. PU Foam Development and Formulation Optimization

**Scheme 2:**
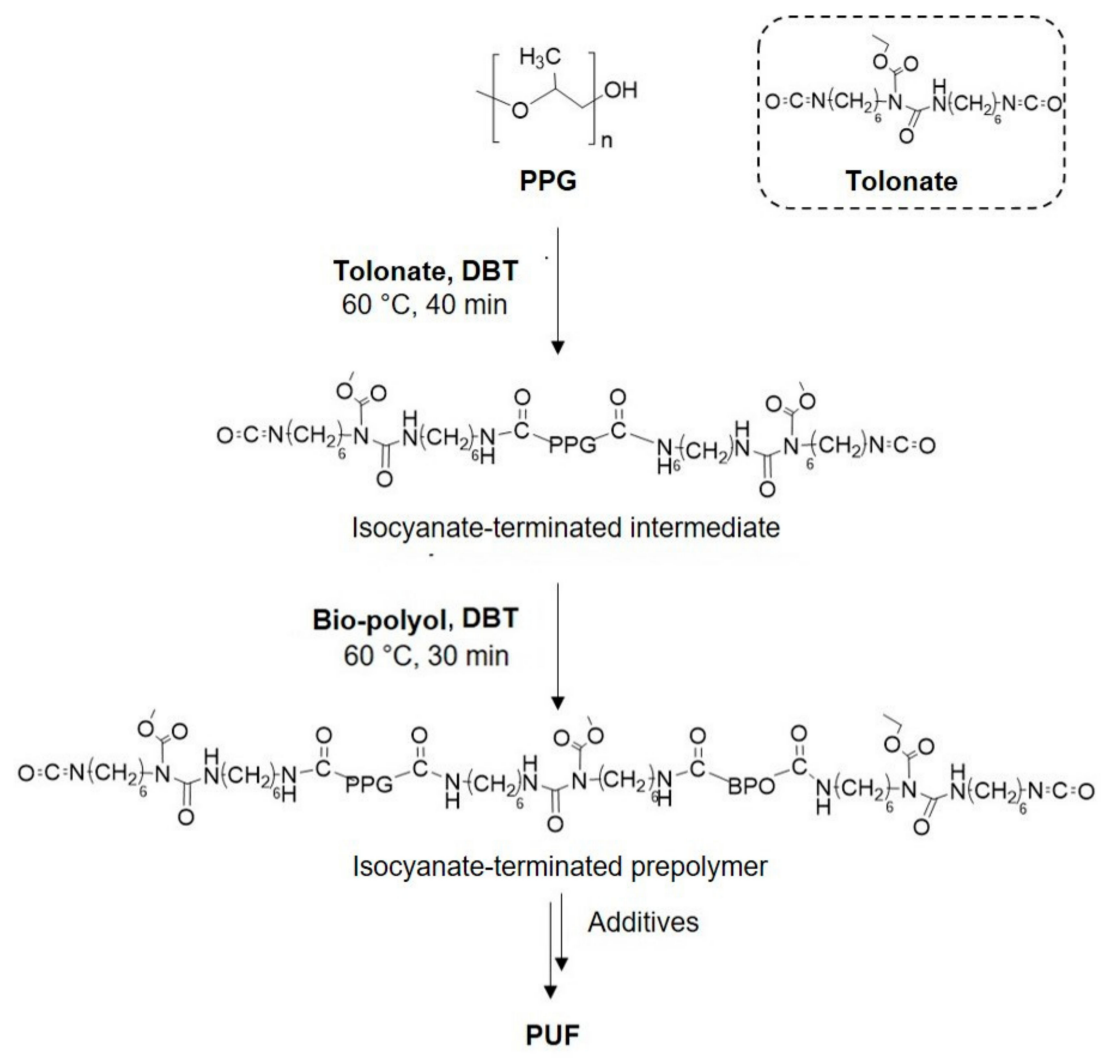
Schematic representation of synthetic routes to prepare foams.

For the synthesis of polyurethane foams, a PU quasi-prepolymer was synthesized by a two-step reaction (Scheme 2). The first step involves the reaction between the polypropylene glycol and a large excess of diisocyanate, giving an isocyanate-terminated intermediate. The second step consists of the reaction of the bio-polyol with the isocyanate-terminated intermediate giving an isocyanate-terminated quasi-prepolymer.

These prepolymers were reacted with some additives to obtain the final PUFs. Representative photos of prepared foams are shown in Figure S7 (Supplementary Materials).

To prepare bio-based PU foams, a polyol from fossil source (PPG) was used in mixture with the synthesized bio-polyols (bio-polyols/PPG weight ratio 2:1) and the silicon surfactant, thus forming a stable emulsion with low water amount. Distilled water was used as chemical blowing agent, and its content kept constant, at 1.5 wt % for all formulations. This amount was chosen because water content would impact both bio-stability of mixtures containing plant-oil-based prepolymer and the chemical composition of the PU foam polymer matrix. A small amount of glycerol (3 wt %) was used to enhance the bio-polyol miscibility with water. The formulations were labeled as Tolo x where x corresponds to the number of the selected bio-polyol. A reference sample (Tolo ref in Table 3) was prepared by using the polyol from fossil sources (PPG) and the same additives.

**Figure 7:**
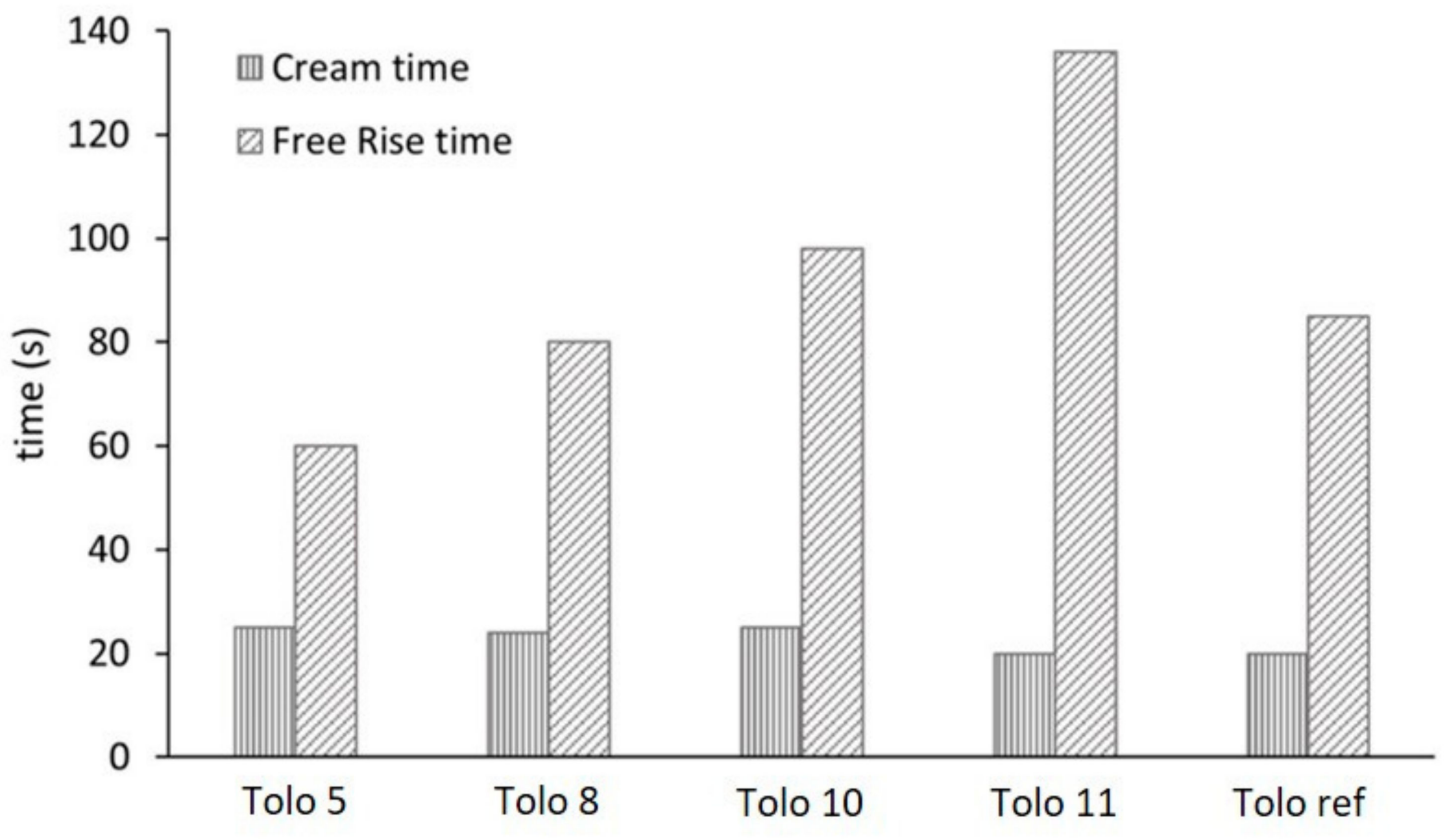
Effect of the different bio-polyols on cream and free rise time.

The foaming process was monitored by recording cream time and rising time. Suitably fast cream time and rising time are important characteristics of such PU systems. The cream time and the free rise time are reported in Figure 7. Similar cream times were observed for all the formulations, regardless of the bio-polyols used. Moving to the rise time, several differences appear. The rising of foams was completed in 60–140 s, with higher rise times achieved with foams from 3-phenyl butyric acid (Tolo 10 and Tolo 11). This aspect could be ascribed to the different polarities of the side chain in bio-polyols coming from 3-phenyl butyric and caprylic acid, respectively, that influence the miscibility with water. Moreover, the reactivity of prepolymers from 3PBA could be influenced by steric hindrance due to the aromatic motive in α position to isocyanate groups. The presence of aromatic side chains in prepolymers leads to longer rise times also in comparison with the reference foam.

The free rise time is strictly associated with density of foams (Table 4), where a slower foaming reaction time leads to higher density.

**Figure 8:**
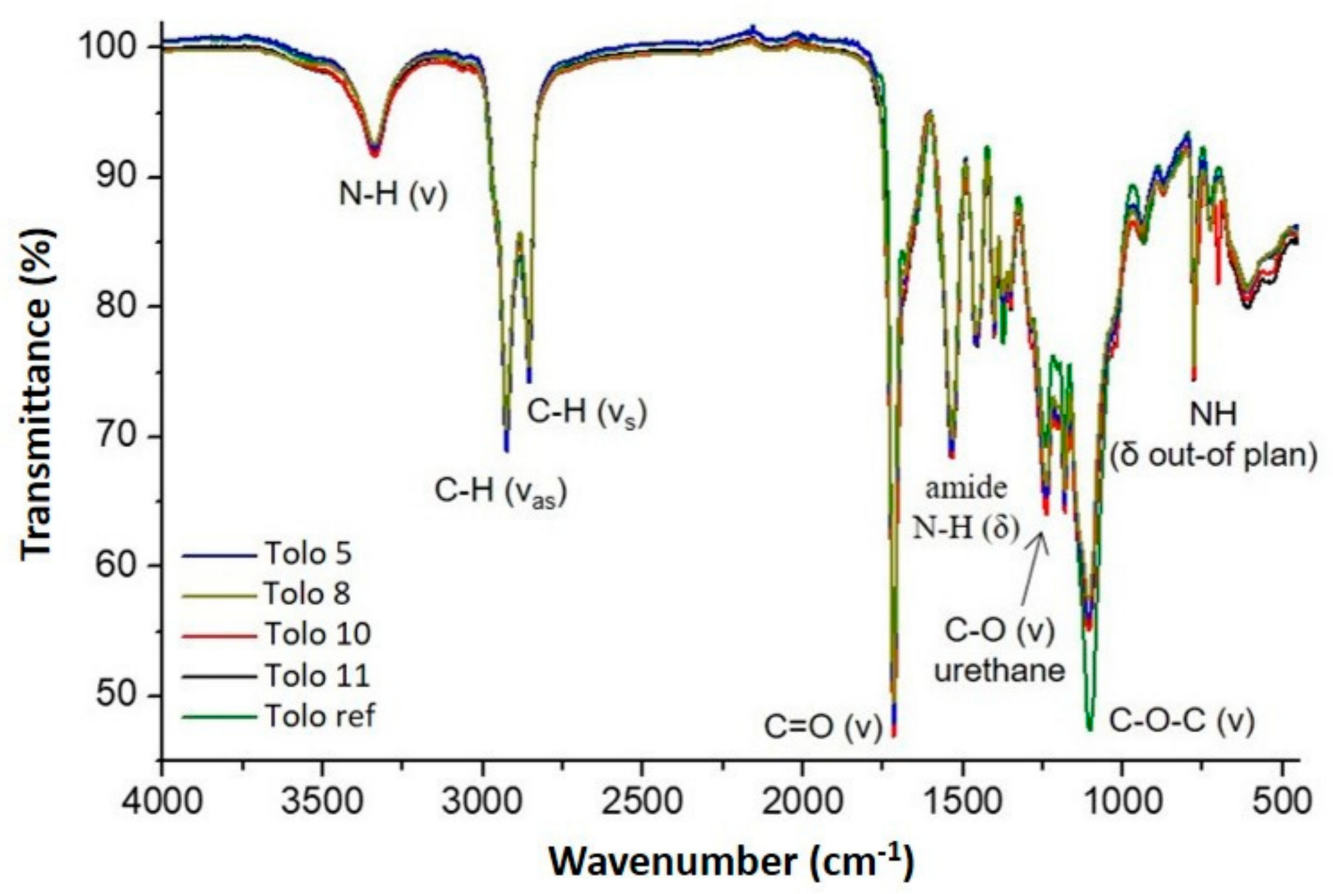
FTIR spectra of the different PUFs.

The PU foams were investigated by FTIR spectroscopy and the spectra are shown in Figure 8. The chemical structure of flexible polyurethane foams consists of alternating of urethane and urea linkage domains [37] and in the range from 1640 to 1780 cm^−1^, the carbonyl stretching vibrations (hydrogen bonded and nonhydrogen bonded) of urethane and urea groups are identified in Figure 8. In detail, all the spectra are characterized by a main absorption peak centered at 1714 cm^−1^ due to hydrogen-bonded urethane ordered C=O groups and carbonyl of free urea close to 1700 cm^−1^ [38]. This strong absorbance shows a shoulder at 1737 cm^−1^ in the spectra from bio-polyols, due to the C=O stretching of ester group in triglycerides. In the range from 1500 to 1560 cm^−1^ the absorption peaks related to the N–H bond of urea and to C–N bond of urea and urethane are observed (the details are reported in Table 1 in Supplementary Materials). The overlapping bands of hydrogen-bonded urea carbonyls, including both monodentate (disordered) and bidentate (ordered), are observed around 1650–1670 cm^−1^ and are indications of hydrogen bonds of differing geometries. Indeed, FTIR analysis shows that the foams prepared with bio-polyols possess the same hard segment structure with respect to those prepared with a standard polyether polyol. All isocyanates groups were reacted with hydroxyl groups to form urethane groups, thus explaining the absence of the peak of the isocyanate group around 2260 cm^−1^ and –OH groups around 3510 cm^−1^. The complete conversion of hydroxyl and isocyanate groups confirms the cross-linking reaction in the employed reaction conditions. The peak at wavelength of 3335 cm^−1^, corresponding to N–H stretching vibration, confirms the reaction between hydroxyl and isocyanates groups, for all PUF spectra. The absorption peaks observed in the range from 1344 to 1460 cm^−1^ are due to –CH bend in alkanes [39] and those at 1100 cm^−1^ are assigned to stretching vibration of ether C–O–C bond in the PPG chain.

The apparent density of PUFs is reported in Table 4, along with strut thickness. The apparent high density of all PUFs was in the range of 79–113 kg/m^3^, due to low amount of blowing agent. The highest density value was obtained for foams from 3-phenyl butyric acid.

The initial viscosity of bio-polyols is one of the most important factors which influence the cellular structure of the resultant foams, affecting the generation and distribution of the cells formed by the blowing agent [40]. The relatively high viscosity of the bio-polyols (Table 2) with respect to the reference PPG (viscosity equal to 0.63 Pa·s) facilitates the formation of the framework of the urethane group in the stage of the gel reaction between the bio-polyol and isocyanate with a low concentration of gel catalyst, resulting in a dense structure and in the high apparent density of the prepared foams, in particular at low water concentration [22,29] and when the aromatic group is inserted in the vegetable oil backbone (Tolo 10 and 11).

**Figure 9:**
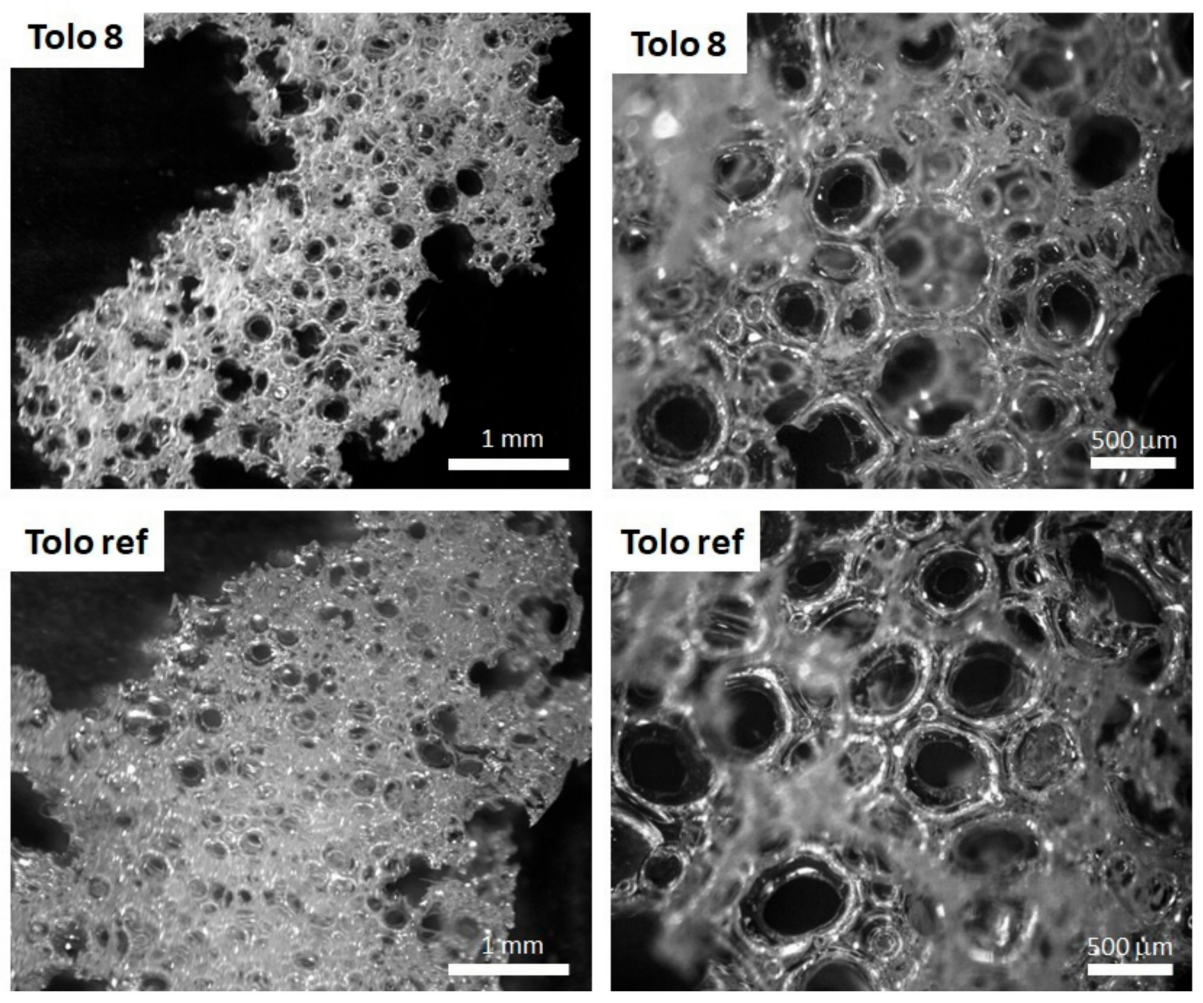
Optical microscope images of Tolo 8 and Tolo ref at different magnifications (10× and 30×).

Figure 9 shows representative images obtained by optical microscope for selected produced foams, Tolo 8 and Tolo ref, at two different magnifications (10× and 30×).

The bio-based foams showed an open cell structure, with a well-developed cavity network, containing interconnected pores (Figure 9 and Figure S8 in the Supplementary Materials). The foam cells have regular and spherical shape and almost uniform size, indicating the isotropic growth of the bubble during the foaming process.

**Figure 10:**
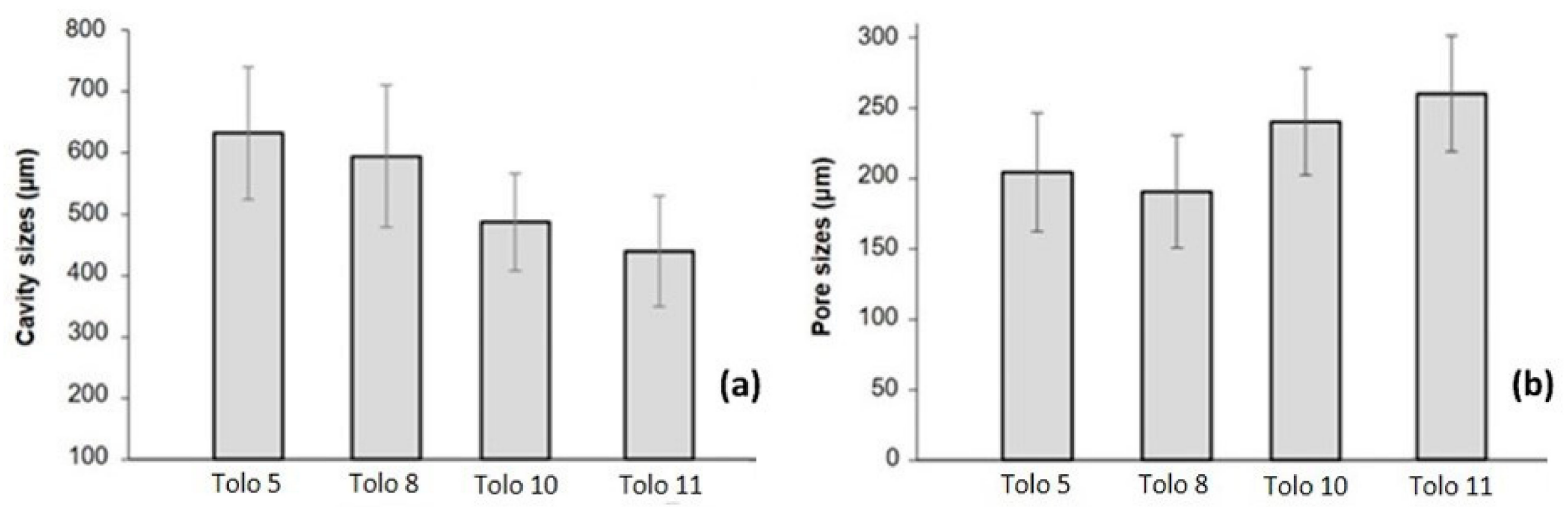
Average cavity (**a**) and pore (**b**) size of the PU foams.

In the foaming process, the slow gelling reaction rate allowed the bubbles to easily escape from the matrix thus forming completely opened cells before they form the firm struts (Figure 9 and Figure S8 in the Supporting Materials). The foaming process produces, especially in bio-based foams, structures with cavities filled by several interconnected pores. The average strut thickness and the average cavity size are closely related to the density of the foams. In Figure 10, the average cavity and pore size distributions for the different studied systems are reported. The foams obtained from 3-phenyl butyric acid (Tolo 10 and Tolo 11) show smaller average cavity size that impacts negatively on density, despite larger pores being present in these foams. As mentioned above, the presence of aromatic moieties that impact on polyol viscosity can create smaller cavities with larger pores surrounded by thicker walls, increasing the strut thickness in comparison with other bio-based foams and negatively impacting on density.

Unlike bio-based foams characterized by a cavity-pore structure, a more uniform structure was noticed in the reference foam, with an average pore size of about 400–600 μm.

**Figure 11:**
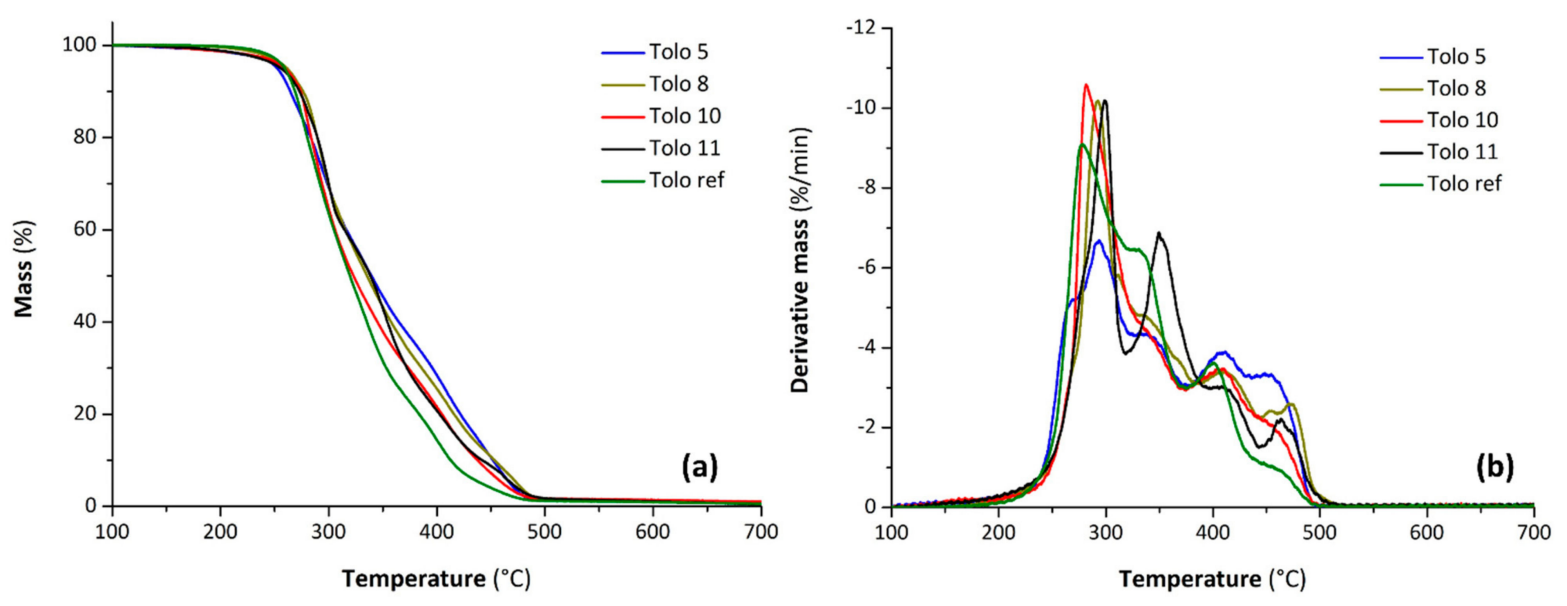
Thermogravimetric analysis (**a**) and derivate thermogravimetry (**b**) curves of PU foams.

Dynamic TGA and DTG curves of the different polyurethanes and the characteristic degradation temperatures are shown in Figure 11 and Table 5, respectively. Thermograms of PU foams show multiple stages of thermal decomposition under inert atmosphere (Figure 11a). In general, the first mass loss stage observed in the range from 200 to 320 °C is associated with ca. 40% of the mass loss and is ascribed to the dissociation of the urethane bonds [41], whereas the successive decomposition stage takes place at higher temperatures (from 320 to 500 °C) and is the result of chain scission in the polyol/bio-polyol structures [42]. In particular, the DTG curve for the latter decomposition step exhibits a broad complex signal constituted by successive degradation events for each sample (Figure 11b). It is worth nothing that at this decomposition stage, the reference foam degrades faster than the foams obtained from bio-polyols; indeed, the mass residue at 400 °C for Tolo ref is close to 15% whereas for Tolo x foam is comprised between 21 and 28%. TGA experimental data including the temperature at which the initial 5% mass loss occurs (*T*_5%_) and the temperatures of maximum rate of mass loss (*T*_max_) determined by main DTG peaks are summarized in Table 5. Considering the *T*_5%_ values (from 253 to 261 °C), the differences in thermal stability of the Tolo x foams appear to be slight: a decreasing OH number of the bio-polyol led to a decreasing number of urethane bonds in the PU foams, and therefore improved thermal stability in the first decomposition stage. Lastly, the differences in *T_max_* values and DTG peak intensities at the second degradation step result are more marked, but there was no obvious trend for these data [43,44].

**Figure 12:**
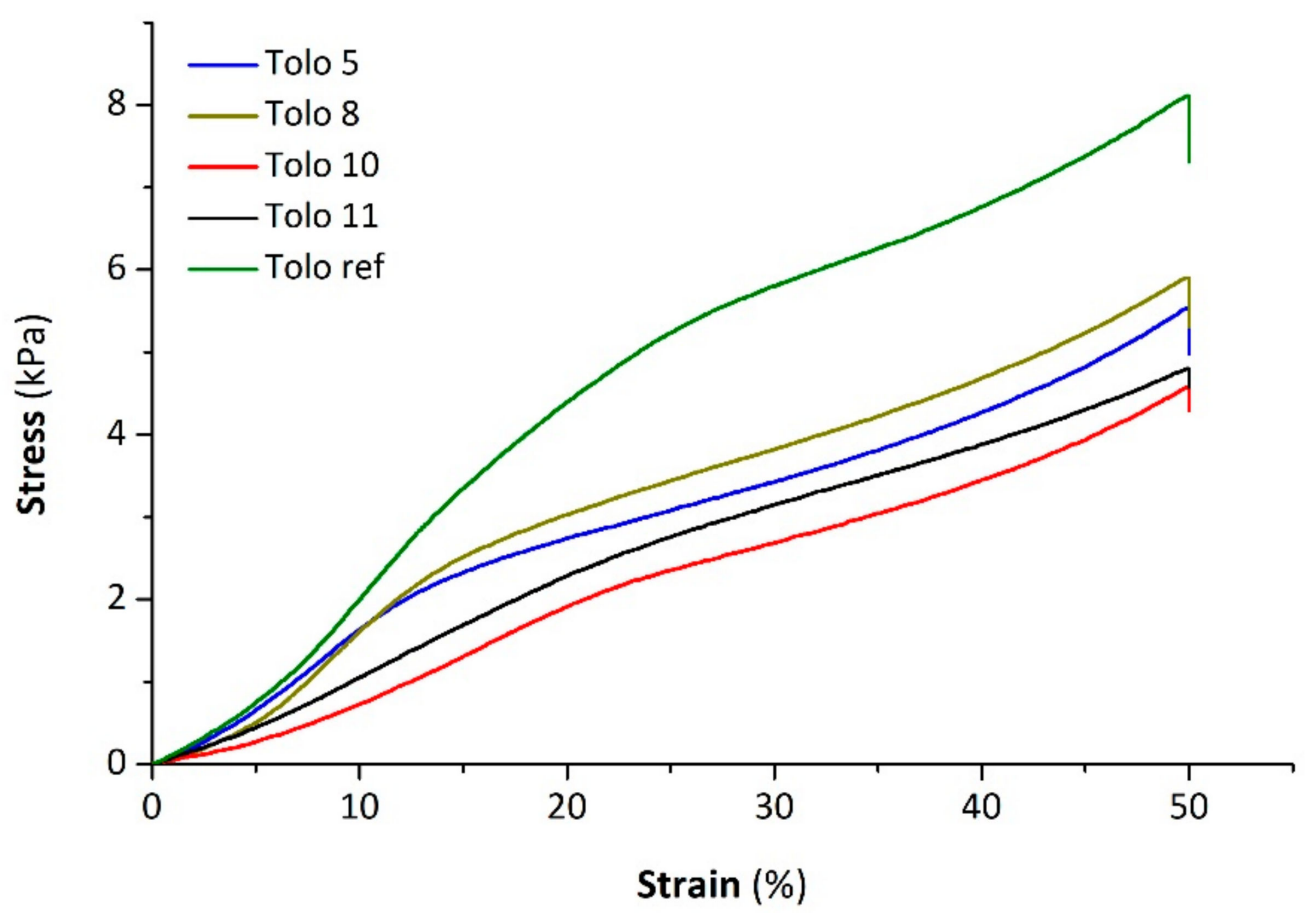
Stress–strain curves of PU foams.

The compressive stress–strain curves of PUFs from different vegetable oils and reference are depicted in Figure 12. Curves of all samples exhibit the typical behavior of polymeric foams characterized by an initial linear portion, due to the elastic deformation of cells, followed by a prominent yield and subsequent progressive increase of the curve related to a partial densification.

The mean values of the mechanical compressive properties of the samples, namely the Young’s modulus and the compression deflection value, calculated as the ratio between the final load and the cross-sectional area of the specimen, are reported in Table 5. The PU foams containing bio-polyols show comparable Young’s moduli and compression deflection values, and are slightly lower than the reference foam. Therefore, the presence of bio-polyols in the samples leads to more flexibility compared to Tolo ref. In particular, Tolo 10 and Tolo 11 show the lowest compression mechanical properties. This behavior is ascribable to larger average pore size. In fact, as highlighted by Gibson et al., the compression strength of ductile cellular solids is inversely proportional to their cell size [45,46]. However, all the samples exhibit an almost complete recovery of the initial size, that denotes the good elastic properties of the prepared flexible PU foams.

**Figure 13:**
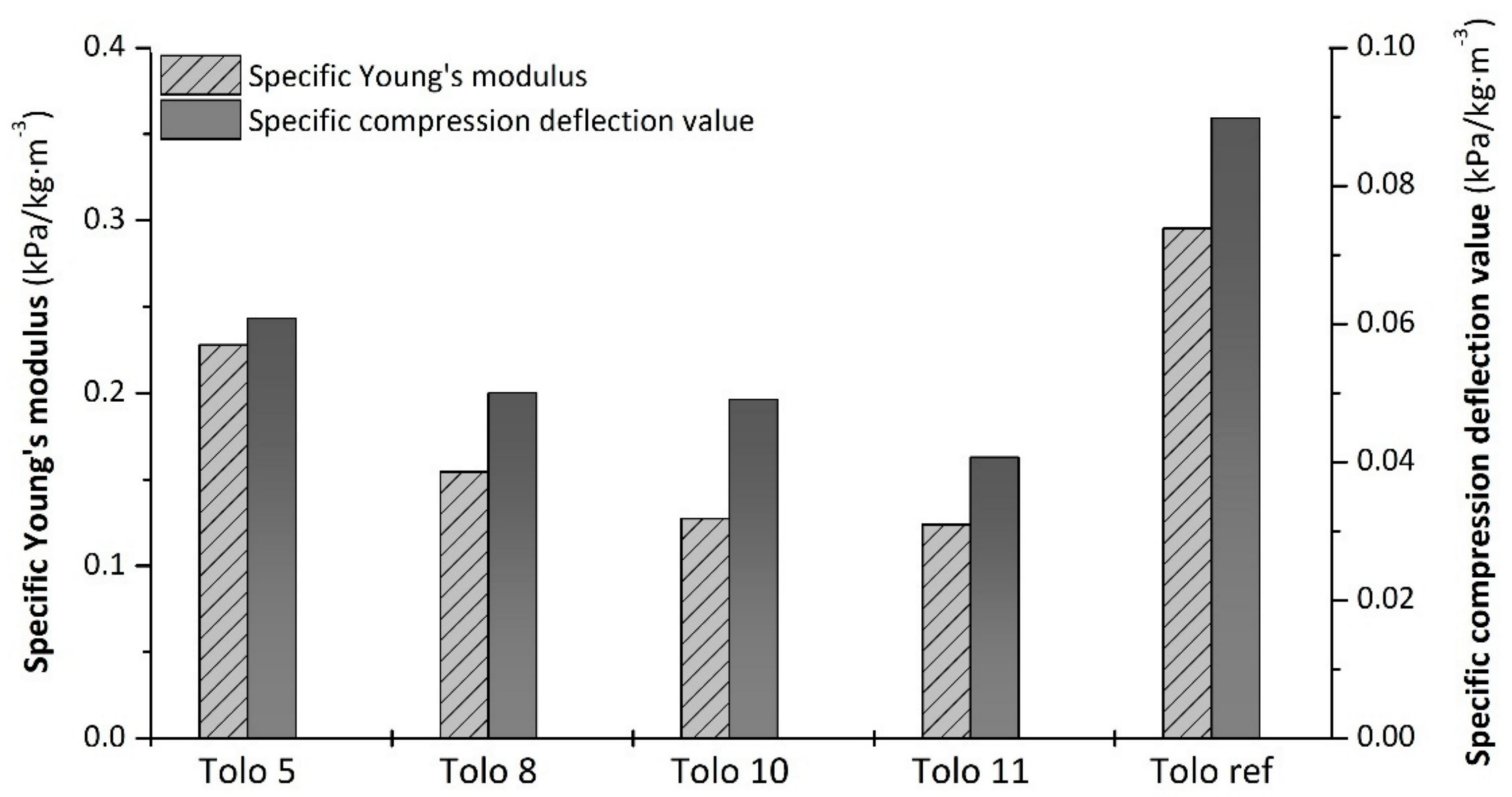
Specific compression properties of PU foams.

Normalizing Young’s moduli and compression deflection values to apparent density is possible to obtain more information about the mechanical behavior of samples. The specific Young’s moduli and compression deflection values, shown in Figure 13, highlight the difference between foams containing bio-polyols and the reference sample. Indeed, lower specific mechanical properties were observed for PUFs from bio-polyols compared to Tolo ref. In particular, the foams Tolo 10 and Tolo 11 from 3-phenyl butyric acid exhibit lower compression mechanical performances.

Moreover, the difference among Tolo ref and the bio-based Tolo x foams could be ascribed to the morphological characteristics. Indeed, the presence of numerous large cavities, connecting smaller pores, seems to give more flexibility, thus impacting on the final mechanical properties of foams from bio-polyols.

## 4. Conclusions

Bio-polyols were prepared from soybean and linseed oil using a solvent-free method. The main characteristics of synthesized bio-polyols, such as hydroxyl value, acid value, viscosity, average molar mass, and polydispersity could be tailored by controlling reaction conditions, which include reaction time, reaction temperature, and molar ratio of epoxidized oil to organic acid. Bio-polyols are a mixture of several oligomerization products as seen from SEC analysis. The use of TEA, a Lewis base catalyst, leads to polyols in an efficient way, minimizing the formation of cross-linked structures and producing polyols with narrow ranges of polydispersity indices.

The obtained bio-polyols with narrow polydispersity indices (1.1–1.4) and relatively low molecular mass of 1600–2400 g/mol were used to prepare flexible polyurethane foams with open cell structures in combination with Tolonate^TM^, an aliphatic bio-based diisocyanate, that represents a promising material for the synthesis of solvent-free PUs with reduced carbon footprint.

The bio-based foams showed an open cell structure, with a well-developed cavity network, containing interconnected pores. The developed polyurethane foams, with an apparent density in the range of 70–115 kg/m^3^, show Young’s moduli and compression deflection values that were slightly lower than those of the reference foam.

## Figures and Tables

**Table 1:** Properties of the bio-polyols.

Run	Epoxidized Oil ^1^	Acid ^2^	Epoxy/ Carboxyl Ratio (eq)	TEA (wt %)	T (°C)	t (h)	*M*_n_^3^ (g/mol)	PDI ^3^	Acid Number ^4^ (mg KOH/g)	OH Number ^5^ (mg KOH/g)
1	ESO	CA	1/0.5	−	170	16	2267	1.6	3	95
2	ESO	CA	1/0.75	−	170	16	2463	1.5	50	132
3	ESO	CA	1/1	−	170	16	2465	1.5	92	166
4	ESO	CA	1/0.5	1	170	16	1951	1.3	6	85
5	ESO	CA	1/0.5	1	130	16	1797	1.1	2	87
6	ESO	CA	1/0.5	1	110	6	1621	1.2	13	106
7	ESO	CA	1/0.5	1	150	6	1776	1.2	15	83
8	ELO	CA	1/0.5	1	150	4	1769	1.4	3	67
9	ESO	3PBA	1/0.5	−	150	16	2400	1.6	37	101
10	ESO	3PBA	1/0.5	1	110	16	1869	1.1	20	93
11	ELO	3PBA	1/0.5	1	130	4	2079	1.2	15	88

^1^ ESO = epoxidized soybean oil, ELO = epoxidized linseed oil; ^2^ CA = caprylic acid, 3PBA = 3-phenyl-butyric acid; ^3^ *M*_n_ = number-average molecular weight and PDI = polydispersity index determined by SEC using polystyrene standards and calculated on the whole distribution of oligomers; ^4^ Determined according to IUPAC 2.201 standard method;^5^ Determined according to ASTM D 4274.

**Table 2:** Properties of selected bio-polyols.

Run	Bio-Polyol Composition ^1^	Viscosity ^2^ (Pa·s)	*M*_n_^3^ (g/mol)	PDI ^3^	Acid Number ^4^ (mg KOH/g)	OH Number ^5^ (mg KOH/g)	OH Functionality ^6^
5	ESO, CA	3.03	1797	1.15	2	87	2.8
8	ELO, CA	4.70	1769	1.36	3	67	2.1
10	ESO, 3PBA	24.26	1869	1.31	20	93	3.1
11	ELO, 3PBA	470.3	2079	1.22	15	88	3.3

^1^ ESO = epoxidized soybean oil, CA = caprylic acid, ELO = epoxidized linseed oil, 3PBA = 3-phenyl butyric acid; epoxy/carboxyl ratio = 1:0.5; ^2^ Measured at 20 °C; ^3^ Determined by SEC using polystyrene standards; ^4^ Determined according to IUPAC 2.201 standard method; ^5^ Determined according to ASTM D 4274; ^6^ Calculated by the formula: *M*_n_* OH_number_/(56.1 * 1000).

**Table 3:** Formulation of the foams used in this study.

	Tolo x	Tolo Ref
Bio-polyol	67	0
PPG	33	100
glycerol	10.8	10.9
water	4.2	4.5
silicone surfactant	7.5	8.9
DBT	0.2	0.2
Isocyanate	294	300
Isocyanante index	100	100

Units are in parts *per* hundred parts (pphp).

**Table 4:** Density and strut thickness of PU (polyurethane) foams.

Sample	Bio-Polyol Composition ^1^	Density ^2^ (kg/m^3^)	Strut Thickness (µm)
Tolo 5	ESO, CA	79 ± 6	35 ± 6
Tolo 10	ESO, 3PBA	110 ± 5	36 ± 10
Tolo 8	ELO, CA	102 ± 2	26 ± 7
Tolo 11	ELO, 3PBA	113 ± 1	37 ± 8
Tolo ref	−	88 ± 10	39 ± 5

^1^ ESO = epoxidized soybean oil, CA = caprylic acid, ELO = epoxidized linseed oil, 3PBA = 3-phenyl butyric acid. ^2^ Determined according to ISO 845:2006.

**Table 5:** Thermal and mechanical properties of PU foams.

	TGA		Compressive Tests	
Sample	*T*_5%_ (°C)	*T*_max_ (°C)	Young’s Modulus (kPa)	50% Compression Deflection Value (kPa)
Tolo 5	253	293, 339, 411, 454	18 ± 4	4.8 ± 1.1
Tolo 8	261	291, 335, 408, 472	17 ± 4	5.5 ± 0.4
Tolo 10	259	281, 409	13 ± 5	5.0 ± 1.8
Tolo 11	256	299, 349, 464	14 ± 1	4.6 ± 0.5
Tolo ref	259	277, 331, 401	26 ± 10	7.9 ± 2.0

## Supplementary Materials

The following are available online at https://www.mdpi.com/2073-4360/13/4/612/s1, Figure S1: Effect of the reaction conditions on the molecular weight of bio-polyols from ESO and caprylic acid, Figure S2: ^13^C-NMR spectrum of bio-polyol from ESO and caprylic acid, Figure S3: ^13^C-NMR spectrum of bio-polyol from ELO and caprylic acid, Figure S4: ^13^C-NMR spectrum of bio-polyol from ESO and 3-phenyl butyric acid, Figure S5: SEC chromatograms of selected bio-polyols, Figure S6: Viscosity of the selected bio-polyols, Figure S7: Representative photos of prepared foam sections, Figure S8. Optical microscope images of Tolo 5, and Tolo 10 and Tolo 11 at different magnifications (10× and 30×), Table S1. Assignments of the main FTIR bands for flexible polyurethane foams.
