# Supplementary material for: Flexible Polyurethane Foams from Epoxidized Vegetable Oils and a Bio-Based Diisocyanate
Source: Polymers (Basel). 2021 Feb 18;13(4):612. doi: 10.3390/polym13040612 (PMC7922077; doi:10.3390/polym13040612)
Supplement: Supplementary file 1 [file polymers-13-00612-s001.pdf]

polymers

MDPI

Supplementary Materials

Flexible Polyurethane Foams from Epoxidized Vegetable Oils and a Bio-based Diisocyanate

Angelica Cifarelli, Laura Boggioni, Adriano Vignali, Incoronata Tritto, Fabio Bertini and Simona Losio*

Institute for Chemical Sciences and Technologies "G. Natta" National Research Council, Via A. Corti 12, 20133 Milan, Italy; angelica.cifarelli@scitec.cnr.it (A.C); laura.boggioni@scitec.cnr.it (L.B.) adriano.vignali@scitec.cnr.it (A.V.); incoronata.tritto@scitec.cnr.it (I.T.); fabio.bertini@scitec.cnr.it (F.B.)
* Correspondence: simona.losio@scitec.cnr.it (S.L.); Tel.: +39-02-23699-369

Citation: Cifarelli, A.; Boggioni, L.; Vignali, A.; Tritto, I.; Bertini, F.; Losio, S. Flexible Polyurethane Foams from Epoxidized Vegetable Oils and a Bio-based Diisocyanate. *Polymers* **2021**, *13*, 612. https://doi.org/10.3390/polym13040612

Received: 29 January 2021
Accepted: 15 February 2021
Published: 18 February 2021

Publisher's Note: MDPI stays neutral with regard to jurisdictional claims in published maps and institutional affiliations.

Copyright: © 2021 by the authors. Licensee MDPI, Basel, Switzerland. This article is an open access article distributed under the terms and conditions of the Creative Commons Attribution (CC BY) license (http://creativecommons.org/licenses/by/4.0/).

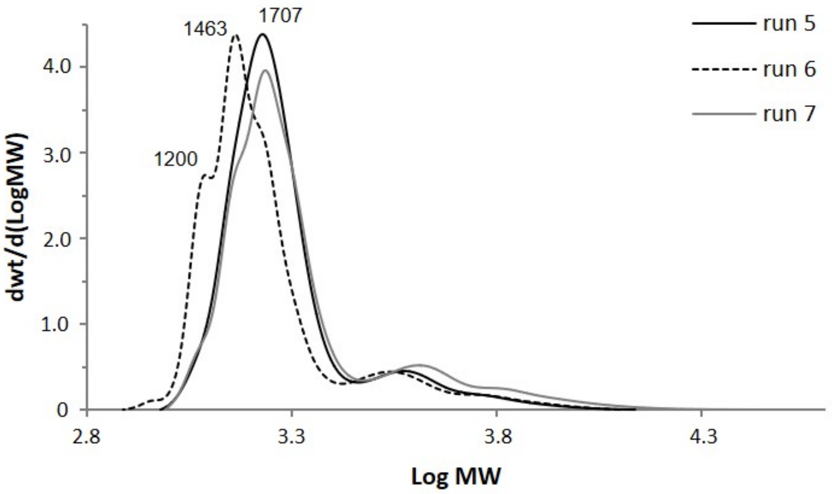

Figure S1. Effect of the reaction conditions on the molecular weight of bio-polyols from ESO and caprylic acid.

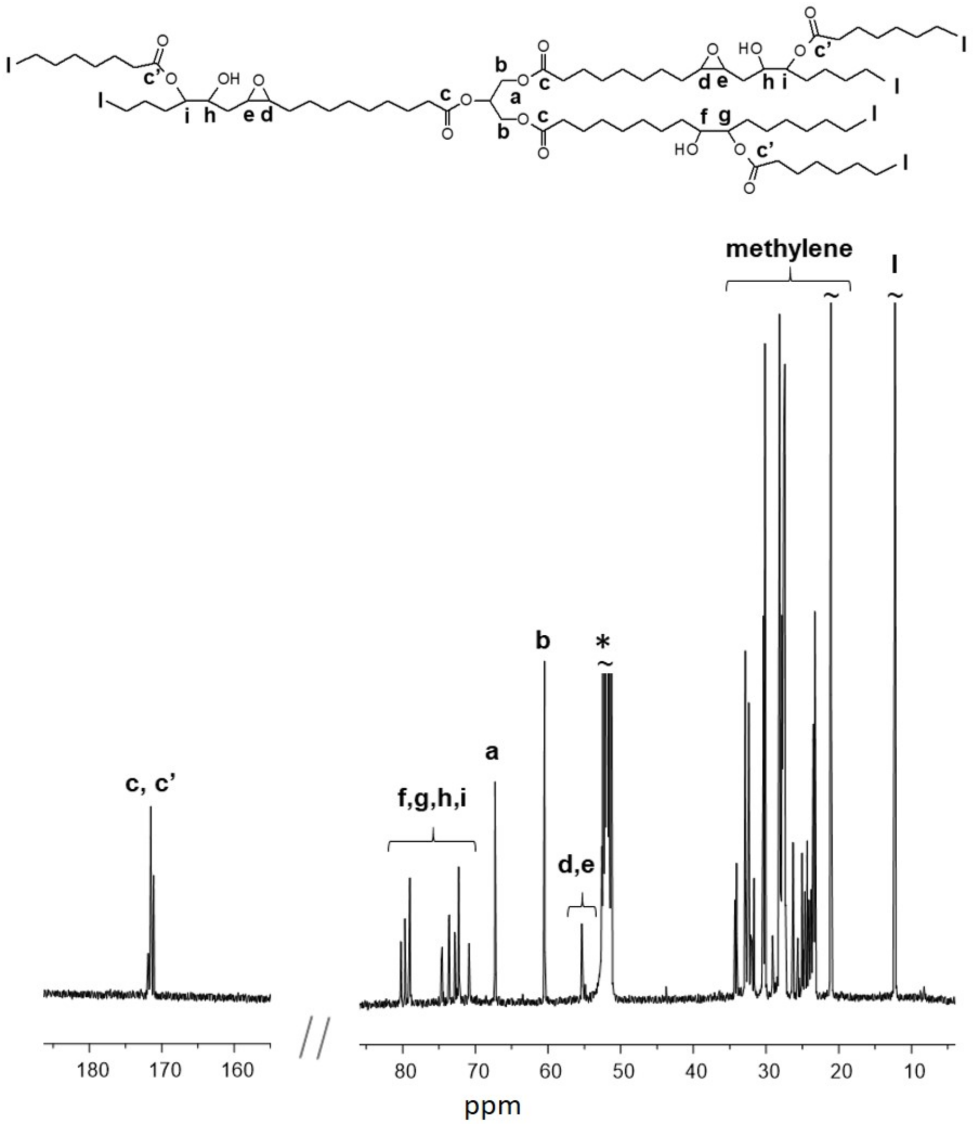

Figure S2. ^{13}C-NMR spectrum of bio-polyol from ESO and caprylic acid (Run 5 in Table 1).

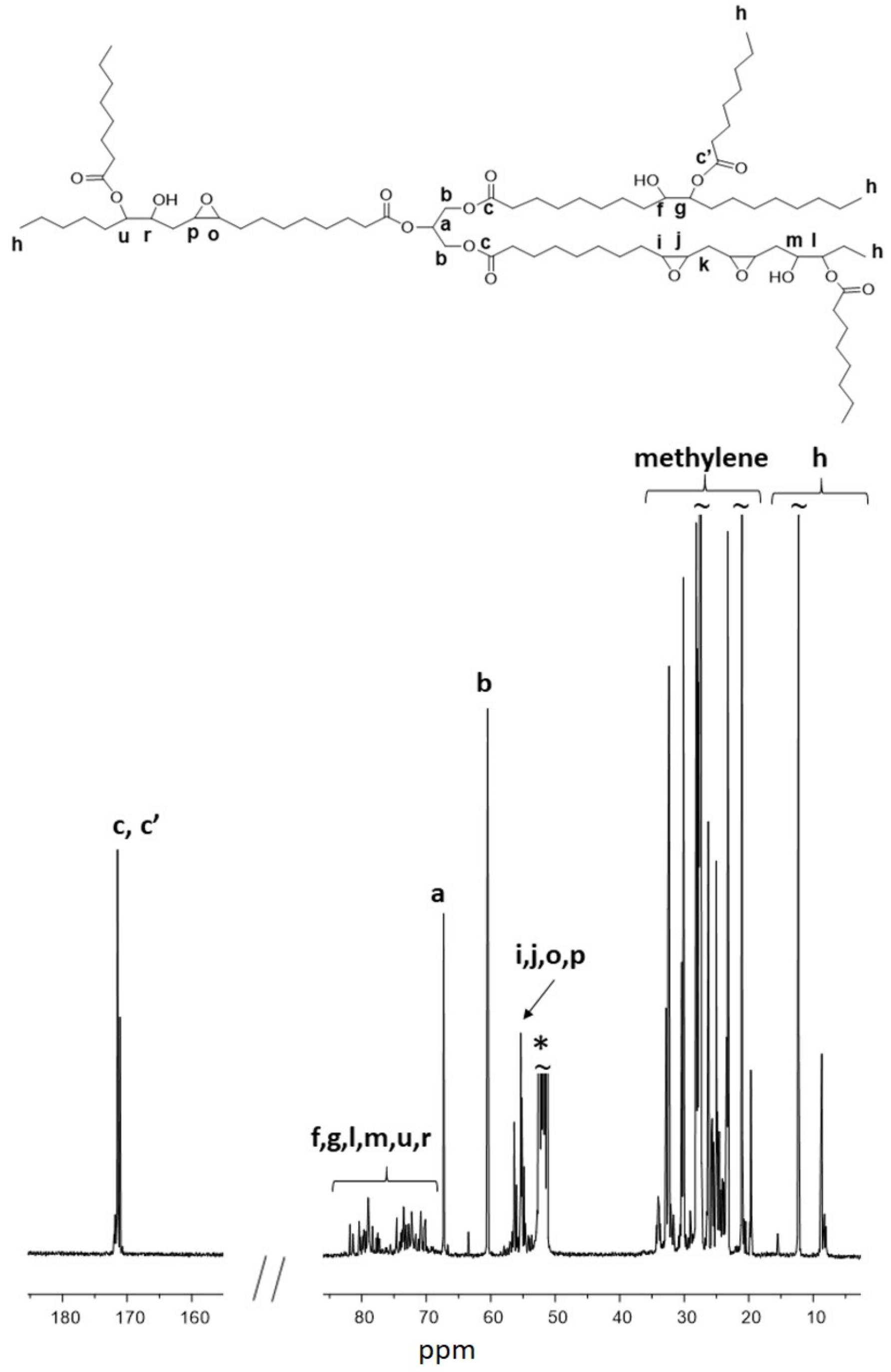

Figure S3. ^{13}C-NMR spectrum of bio-polyol from ELO and caprylic acid (Run 8 in Table 1).

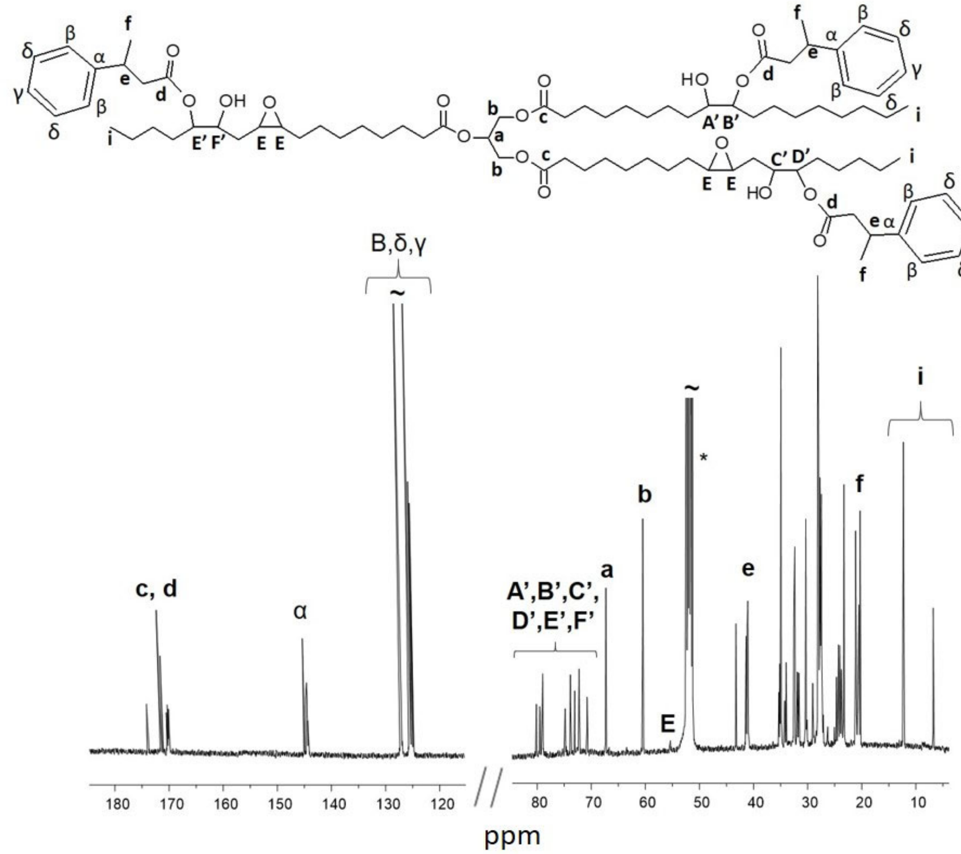

Figure S4. ^{13}C-NMR spectrum of bio-polyol from ESO and 3-phenyl butyric acid (Run 10 in Table 1).

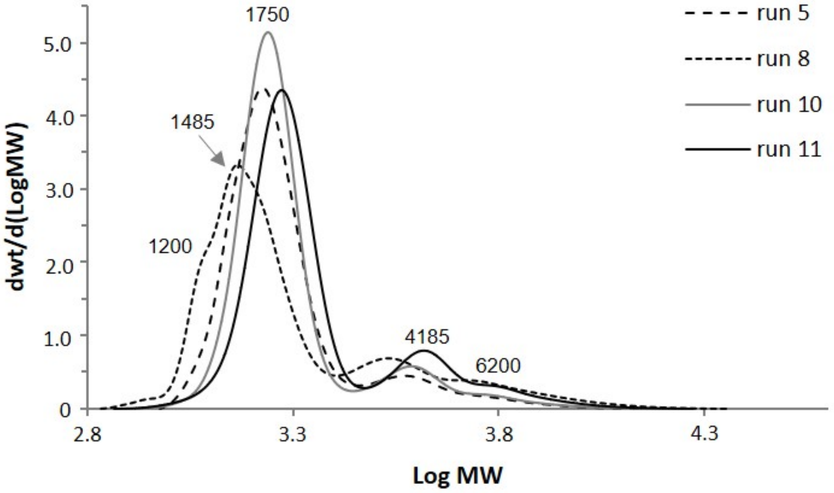

Figure S5. SEC chromatograms of selected bio-polyols.

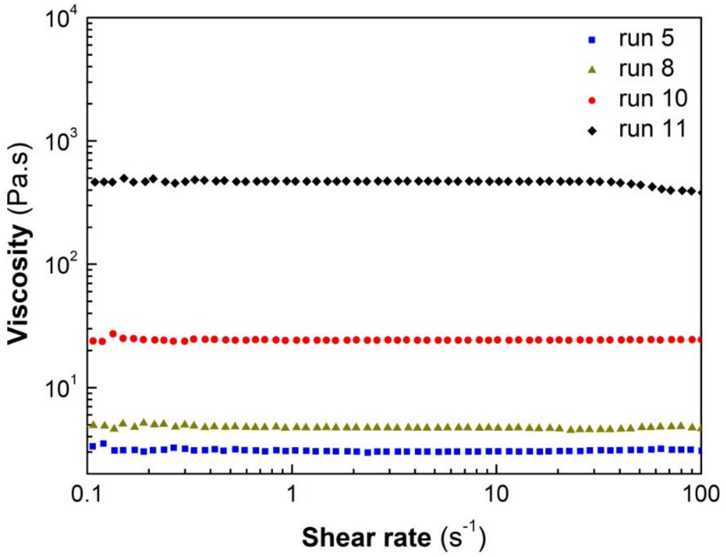

Figure S6. Viscosity of the selected bio-polyols.

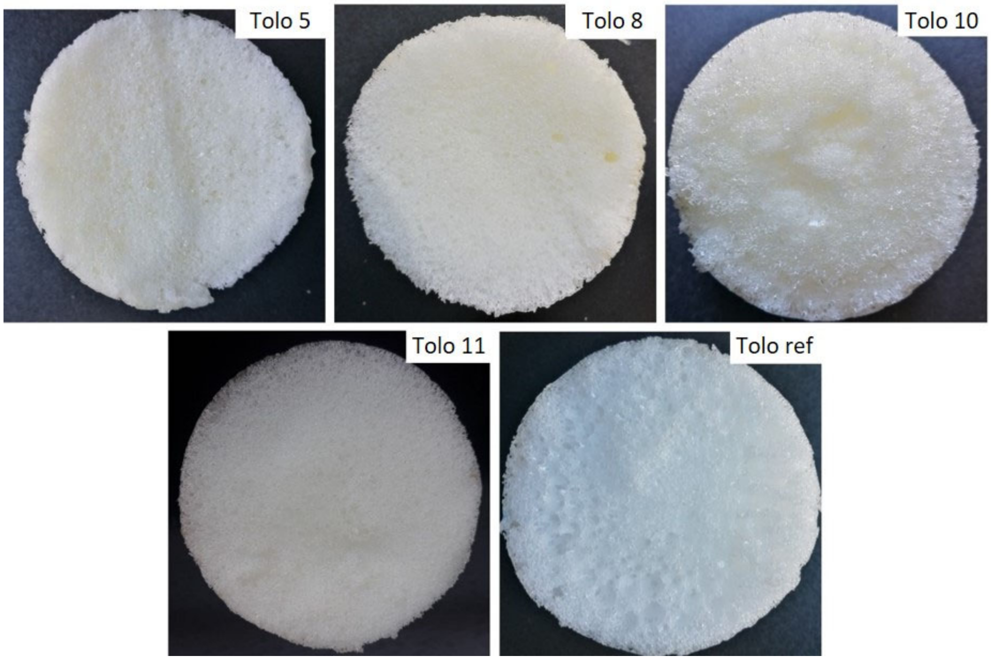

Figure S7. Representative photos of prepared foam sections.

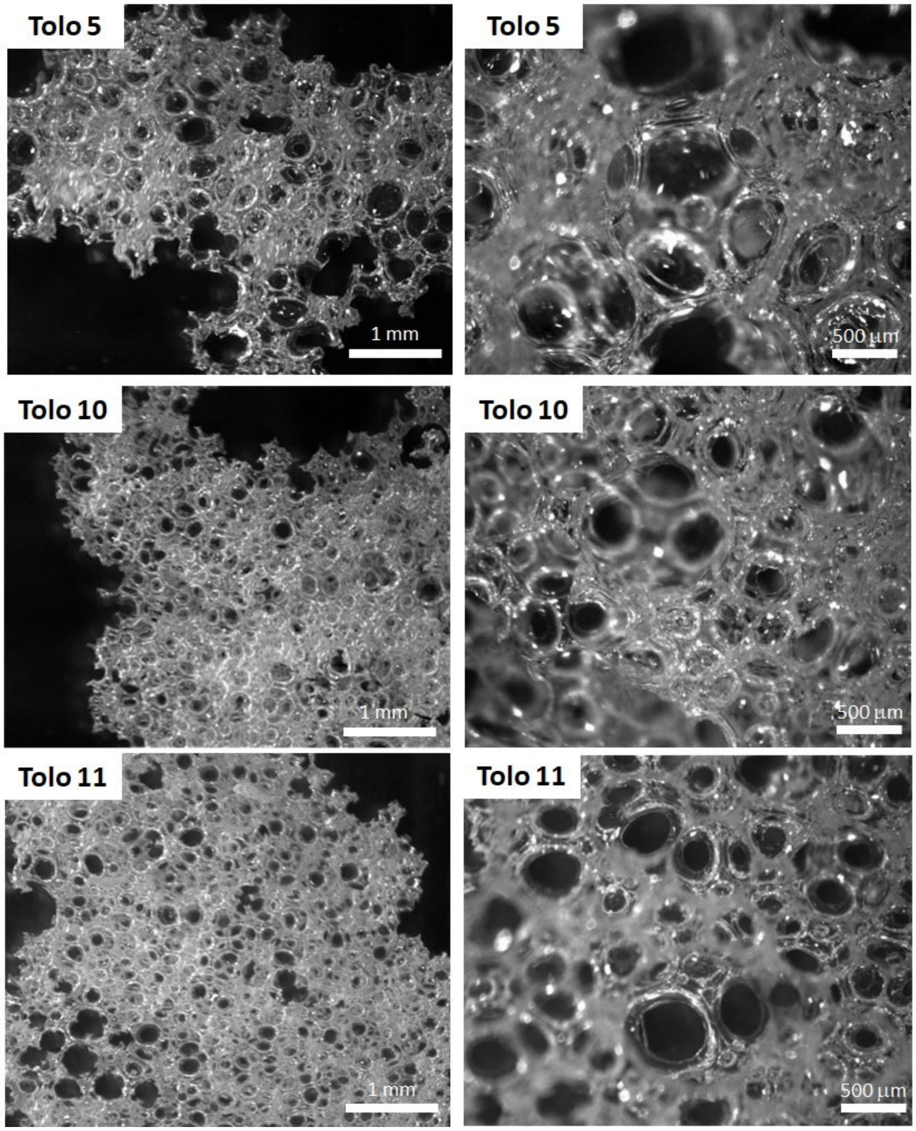

Figure S8. Optical microscope images of Tolo 5, and Tolo 10 and Tolo 11 at different magnifications (10× and 30×).

Table S1. Assignments of the main FTIR bands for flexible polyurethane foams.

Assignment	Group Assignment	Wavenumber (cm^{-1})				
		Tolo 5	Tolo 8	Tolo 10	Tolo 11	Tolo ref
ν_{st} C-O-C	Ether bond (PPG chain)	1107	1106	1107	1106	1106
ν_{st} N-H	Urea	1502	1502	1503	1502	1501
ν_{st} C-H & δ-N-H	Urea	1534	1532	1533	1533	1534
δ-C-N	Urethane	~1561	~1562	~1562	~1563	~1562
ν_{st} C=O	Urea bidentate	~1639	~1639	~1639	~1641	~1639
ν_{st} C=O	Urea monodentate	~1679	~1678	~1676	~1679	~1679
ν_{st} C=O	Urethane-H bonded & Free Urea	1714	1715	1714	1714	1714
ν_{st} C=O	Polyol residues (ester group)	1738	1736	1737	1738	-
